# Supplementary material for: Differences in the functional brain architecture of sustained attention and working memory in youth and adults
Source: PLoS Biol. 2022 Dec 21;20(12):e3001938. doi: 10.1371/journal.pbio.3001938 (PMC9815648; doi:10.1371/journal.pbio.3001938)
Supplement: S1 Text — Fig A in S1 Text. Task accuracy (top) and network strength (bottom) as a function of stimulus types. Fig B in S1 Text. Results of the mediation analyses relating block-by-block performance in the 2-back task to stimulus type, with fluctuations in working memory network strength as a mediator. Fig C in S1 Text. Working memory network strength, but not sustained attention network strength, was predictive of children’s subsequent memory task performance. Fig D in S1 Text. Relationship between cognitive composite network strength and cognitive composite scores in the ABCD sample in all iterations of leave-one-site out combined. Fig E in S1 Text. Relationship between cognitive composite network strength and cognitive composite scores in the ABCD sample in each site separately is shown with blue points on permutation null distributions (violins). Table A in S1 Text. Model predicting individual differences in 0-back and 2-back task accuracy from cognitive composite network strength in the ABCD Study sample. Table B in S1 Text. Model predicting individual differences in 0-back and 2-back task accuracy from cognitive composite and sustained attention network strength in the ABCD Study sample. Table C in S1 Text. Model predicting intra-individual differences in 0-back and 2-back task accuracy from youth cognitive composite network strength in the ABCD Study sample. Fig F in S1 Text. Relationship between sustained attention network strength and n-back accuracy in the adult HCP sample. Table D in S1 Text. Model predicting individual differences in 0-back and 2-back task accuracy from sustained attention network strength in the HCP sample. Table E in S1 Text. Model predicting intra-individual differences in 0-back and 2-back task accuracy from sustained attention network strength and block type in the HCP sample. Fig G in S1 Text. The instruction and first 3 trials in a block of 0-back task (top, example from Places block type) and 2-back task (bottom, example from Positive Face [file pbio.3001938.s001.docx]

**Title: Differences in the functional brain architecture of sustained attention and working memory in youth and adults**

Authors: Omid Kardan^1,2^*, Andrew J. Stier^1^, Carlos Cardenas-Iniguez^1^, Kathryn E. Schertz^1^, Julia C. Pruin^1^, Yuting Deng^1^, Taylor Chamberlain^1^, Wesley J. Meredith^3^, Xihan Zhang^1,4^, Jillian E. Bowman^1^, Tanvi Lakhtakia^1^, Lucy Tindel^1^, Emily W. Avery^4^, Qi Lin^4^, Kwangsun Yoo^4^, Marvin M. Chun^4^, Marc G. Berman^1^, & Monica D. Rosenberg^1^*

^1^University of Chicago

^2^University of Michigan

^2^University of California, Los Angeles

^3^Yale University

*Correspondence: omidk@med.umich.edu and mdrosenberg@uchicago.edu

**S1. Appendix: Supplementary Analyses and Material**

**Stimulus type does not explain within-subject relationships between network strength and behavior.** As demonstrated in Study 1.2, the strength of the sustained attention and working memory networks is related to sustained attention and working memory performance within-subject even when adjusting for stimulus type. To what degree are fluctuations in behavior and functional connectivity driven by internal factors (e.g., fluctuations in intrinsic cognitive and attentional states) versus external factors (such as the characteristics of the task itself)? In other words, is the within-subject variance in *n*-back performance captured by the connectivity models driven by task design or endogenous sources? To answer this question, we examined the relationship of the *n*-back stimulus types with changes in task accuracy and changes in the sustained attention and working memory network strength. When applicable, we then conducted mediation analyses to assess the extent to which changes in networks strength mediated the effects of stimulus type on block-wise task accuracy.

We first tested whether 0-back accuracy differed as a function of stimulus type (neutral face, positive face, negative face, place). Five of the six pairwise contrasts were significant, such that 0-back accuracy was higher for neutral vs. negative faces, neutral faces vs. places, positive vs. negative faces, positive faces vs. places, and negative faces vs. places (all *p*_adj_ < .001; Tukey’s T test). Next, we calculated pairwise differences in sustained attention network strength during 0-back blocks with different stimulus types. One pairwise difference was significant, such that sustained attention network strength during place blocks was *higher* than sustained attention network strength during negative face blocks (*p*_adj_ = .026). This demonstrates that the within-subject relationship between sustained attention network strength and 0-back accuracy *cannot* be driven by stimulus type because the only stimulus-driven associations between within-subject changes in sustained attention network strength and 0-back accuracy (places vs. negative faces) are in opposite directions (**Fig A in S1 Text**, left column).

In the 2-back task, there were three significant pairwise differences in accuracy: better performance in neutral face vs. place, positive face vs. place, and negative face vs. place blocks (all *p*_adj_ < .001). Only one pairwise difference in working memory network strength was significant, such that strength was higher during neutral face than place blocks (*p*_adj_ =.005; **Fig A in S1 Text**, right column).

Because both 2-back accuracy and working memory network strength were higher during neutral face than place blocks, we conducted a mediation analysis to test whether the relationship between stimulus type (neutral face vs. place) and 2-back accuracy was mediated by changes in working memory network strength. Results demonstrate that changes in working memory network strength partially mediated the effect of stimulus type on block-wise 2-back accuracy (significant differences between total effect c and direct effect c’ in the mediation; c-c’ = .004, p = .004; **Fig B in S1 Text**). The proportion of mediated effect was modest (about 1% of the total effect).

Together these findings suggest that changes in sustained attention and working memory network strength are driven to a greater degree by intrinsic fluctuations than stimulus characteristics, and that within-subject associations between network strength and task accuracy are, to a large extent, independent of stimulus type in this task.


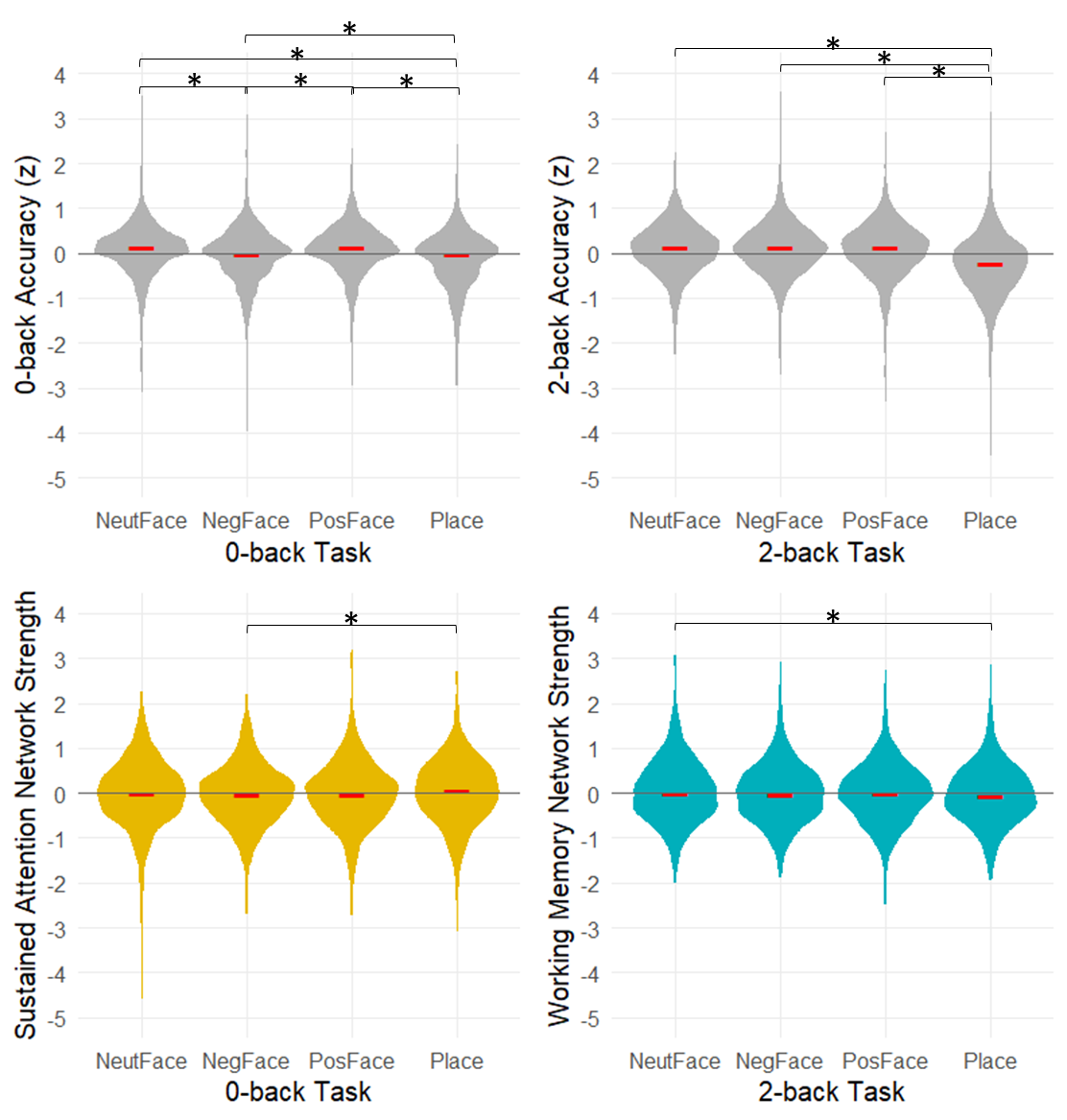


**Fig A.** Task accuracy (top) and network strength (bottom) as a function of stimulus types. Red dash indicates median, and * indicates *p*_adj_ < .05 for pairwise comparisons. The data for this figure are available at NDA study 1849 [10.15154/1528288](http://dx.doi.org/10.15154/1528288).


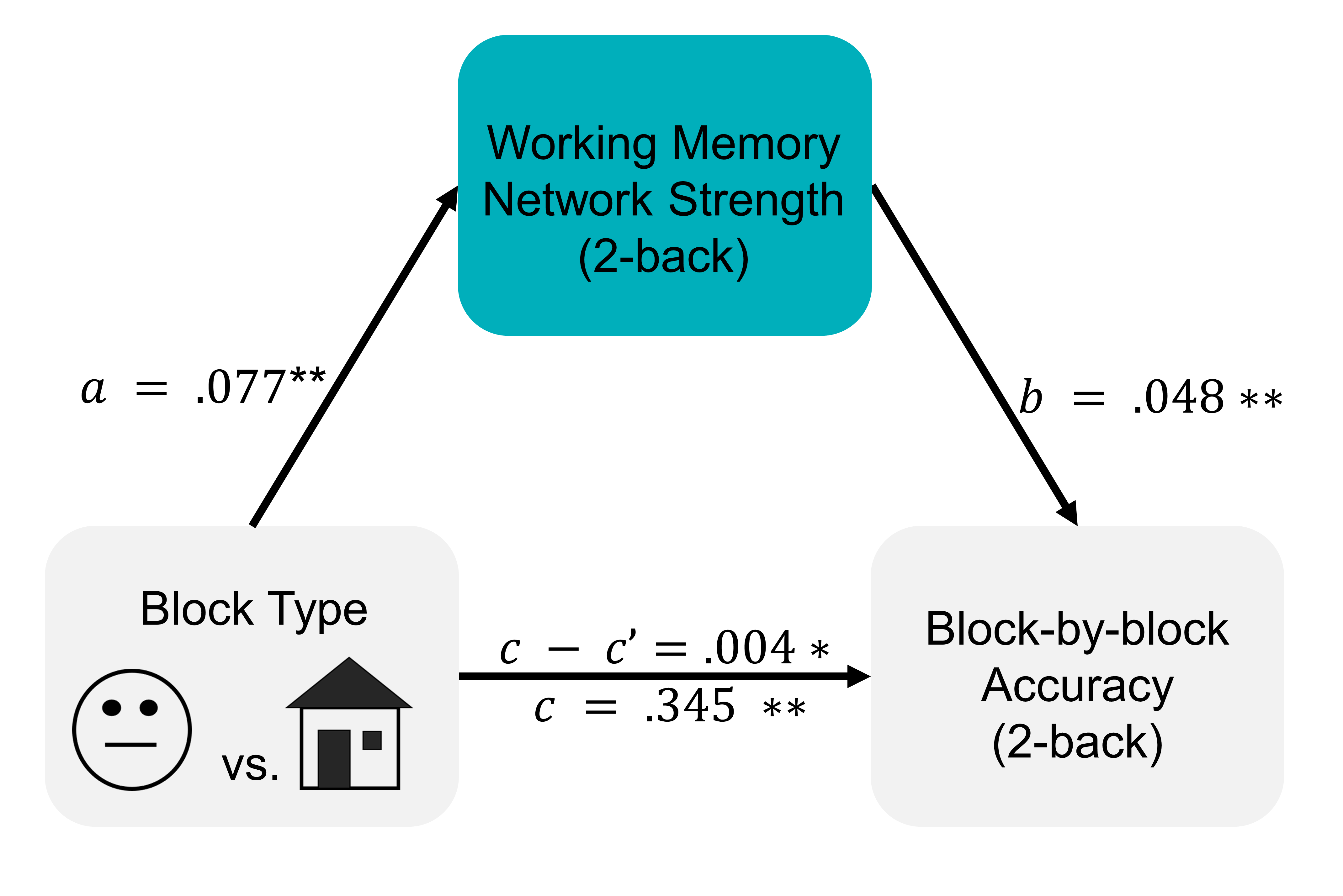


**Fig B.** Results of the mediation analyses relating block-by-block performance in the 2-back task to stimulus type, with fluctuations in working memory network strength as a mediator. The total vs. mediated effects are shown as c and c - c’, respectively. All coefficients are adjusted for block-wise motion.


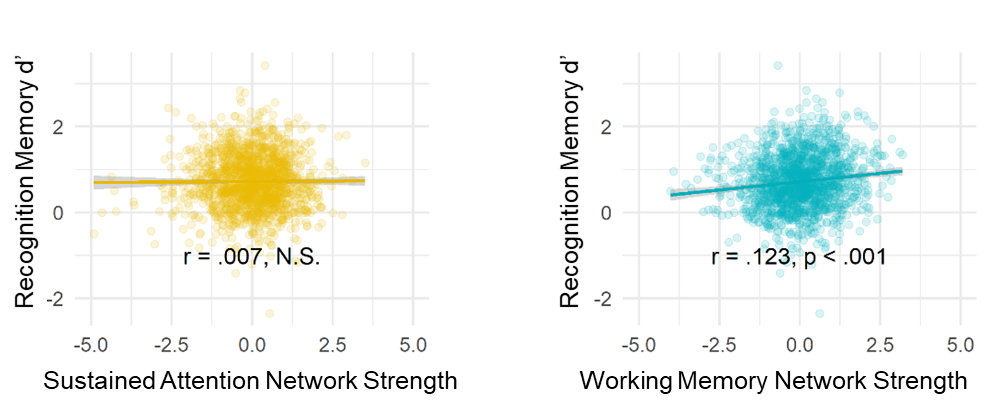


**Fig C.** Working memory network strength, but not sustained attention network strength, was predictive of children’s subsequent memory task performance. The data for this figure are available at NDA study 1849 [10.15154/1528288](http://dx.doi.org/10.15154/1528288).


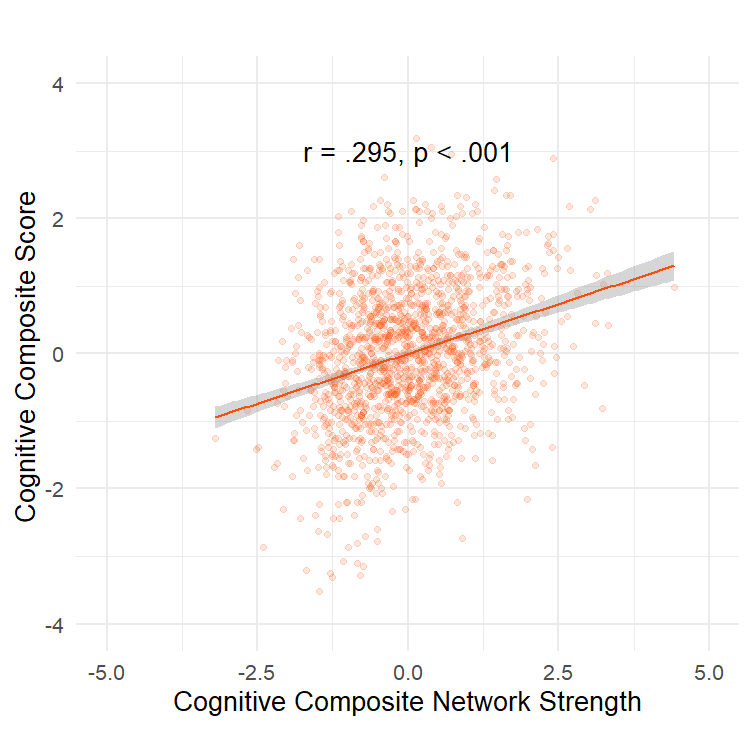


**Fig D.** Relationship between cognitive composite network strength and cognitive composite scores in the ABCD sample in all iterations of leave-one-site out combined. The data for this figure are available at NDA study 1849 [10.15154/1528288](http://dx.doi.org/10.15154/1528288).

**
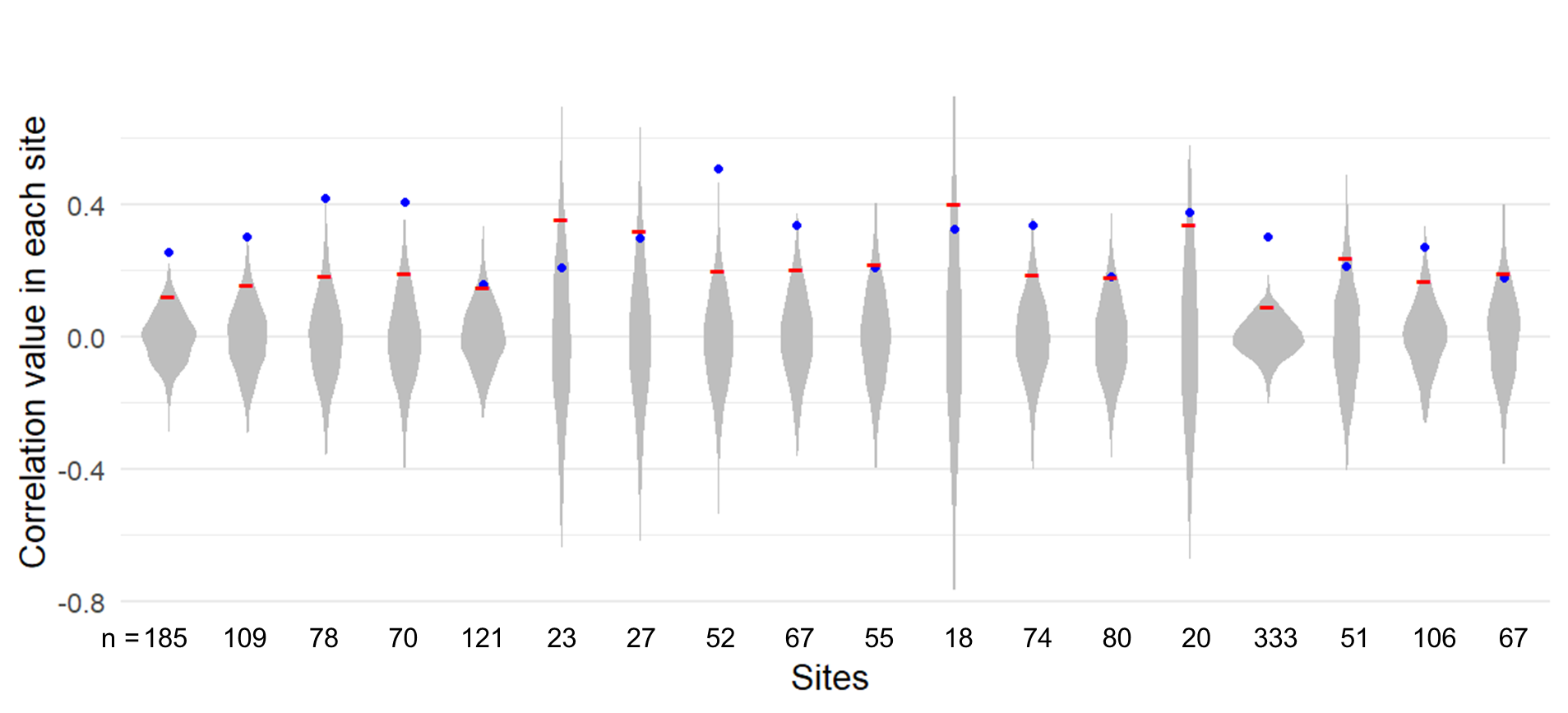
**

**Fig E.** Relationship between cognitive composite network strength and cognitive composite scores in the ABCD sample in each site separately is shown with blue points on permutation null distributions (violins). The red dash indicates the 95^th^ percentile of the null distribution values and the number below each violin indicates the number of participants retained in the study (n) from the corresponding site. Two sites with Philips scanner data and one site with *n* = 3 after motion exclusions were not included in the study analyses (see *Methods*). The data for this figure are available at NDA study 1849 [10.15154/1528288](http://dx.doi.org/10.15154/1528288).

|  | | **0-back Accuracy** | | | | |  | **2-back Accuracy** | | | |
| --- | --- | --- | --- | --- | --- | --- | --- | --- | --- | --- | --- |
| *Predictors* | | *Beta* | *CI* | | *t* | *p* |  | *Beta* | *CI* | *t* | *p* |
| *(Intercept)* | | 0.02 | | [-0.07, 0.11] | 0.38 | 0.707 |  | 0.12 | [0.03, 0.21] | 2.67 | **0.012** |
| *Age* | | 0.14 | | [0.09, 0.19] | 5.75 | **<0.001** |  | 0.13 | [0.08, 0.17] | 5.22 | **<0.001** |
| *Female* | | -0.04 | | [-0.18, 0.02] | -0.89 | 0.374 |  | -0.23 | [-0.32, -0.13] | -4.75 | **<0.001** |
| *Motion* | | -0.05 | | [-0.10, 0.00] | -1.93 | 0.054 |  | -0.12 | [-0.16, -0.07] | -4.83 | **<0.001** |
| Cognitive Composite network strength | | 0.19 | | [0.14, 0.24] | 7.57 | **<0.001** |  | 0.27 | [0.22, 0.31] | 10.86 | **<0.001** |
|  | |  | |  |  |  |  |  |  |  |  |
| **Random Effects**  **(Site)** | |  | |  |  |  |  |  |  |  |  |
|  | ICC | 0.01_site_ | | | | |  | 0.01_site_ | | | |
| Observations | | 1545 | | | | |  | 1545 | | | |
| Marginal R^2^ / Conditional R^2^ | | 0.071 / 0.083 | | | | |  | 0.136 / 0.148 | | | |

**Table A.** Model predicting individual differences in 0-back and 2-back task accuracy from cognitive composite network strength in the ABCD Study sample.

|  | | **0-back Accuracy** | | | | |  | **2-back Accuracy** | | | |
| --- | --- | --- | --- | --- | --- | --- | --- | --- | --- | --- | --- |
| *Predictors* | | *Beta* | *CI* | | *t* | *p* |  | *Beta* | *CI* | *t* | *p* |
| *(Intercept)* | | 0.03 | | [-0.06, 0.12] | 0.66 | 0.522 |  | 0.12 | [0.03, 0.21] | 2.69 | **0.011** |
| *Age* | | 0.12 | | [0.08, 0.17] | 5.03 | **<0.001** |  | 0.13 | [0.08, 0.17] | 5.18 | **<0.001** |
| *Female* | | -0.06 | | [-0.15, 0.04] | -1.14 | 0.255 |  | -0.23 | [-0.32, -0.13] | -4.75 | **<0.001** |
| *Motion* | | -0.03 | | [-0.08, 0.01] | -1.37 | 0.172 |  | -0.12 | [-0.16, -0.07] | -4.77 | **<0.001** |
| Cognitive Composite network strength | | 0.21 | | [0.16, 0.26] | 8.20 | **<0.001** |  | 0.27 | [0.22, 0.32] | 10.64 | **<0.001** |
| Sustained attention network strength | | 0.19 | | [0.14, 0.23] | 7.59 | **<0.001** |  | 0.01 | [-0.03, 0.06] | 0.56 | 0.573 |
|  | |  | |  |  |  |  |  |  |  |  |
| **Random Effects**  **(Site)** | |  | |  |  |  |  |  |  |  |  |
|  | ICC | 0.01_site_ | | | | |  | 0.01_site_ | | | |
| Observations | | 1545 | | | | |  | 1545 | | | |
| Marginal R^2^ / Conditional R^2^ | | 0.106 / 0.117 | | | | |  | 0.136 / 0.148 | | | |

**Table B.** Model predicting individual differences in 0-back and 2-back task accuracy from cognitive composite and sustained attention network strength in the ABCD Study sample.

|  | | **0-back Accuracy** | | | |  | **2-back Accuracy** | | | |
| --- | --- | --- | --- | --- | --- | --- | --- | --- | --- | --- |
| *Predictors* | | *Beta* | *CI* | *t* | *p* |  | *Beta* | *CI* | *t* | *p* |
| *(Intercept)* | | 0.07 | [0.02, 0.12] | 2.83 | **0.005** |  | 0.03 | [-0.02, 0.08] | 1.23 | 0.219 |
| *Block Motion* | | -0.03 | [-0.06, -0.01] | -3.20 | **0.001** |  | -0.02 | [-0.04, -0.00] | -2.11 | **0.035** |
| *Block Type:* | |  |  |  |  |  |  |  |  |  |
|  | *Negative Face* | -0.13 | [-0.18, -0.09] | -5.80 | **<0.001** |  | -0.00 | [-0.05, 0.04] | -0.13 | 0.896 |
|  | *Positive Face* | 0.01 | [-0.03, 0.06] | 0.47 | 0.635 |  | -0.03 | [-0.07, 0.02] | -1.10 | 0.269 |
|  | *Place* | -0.23 | [-0.27, -0.18] | -9.99 | **<0.001** |  | -0.37 | [-0.41, -0.32] | -16.18 | **<0.001** |
| *Run 2 – Run 1* | | 0.03 | [-0.01, 0.06] | 1.38 | 0.167 |  | 0.16 | [0.12, 0.20] | 8.54 | **<0.001** |
| Cognitive Composite network strength | | 0.05 | [0.03, 0.07] | 5.37 | **<0.001** |  | 0.08 | [0.06, 0.10] | 8.33 | **<0.001** |
|  | |  |  |  |  |  |  |  |  |  |
| **Random Effects**  **(Subject)** | |  |  |  |  |  |  |  |  |  |
|  | ICC | 0.40_subs_ | | | |  | 0.38_subs_ | | | |
| Observations (blocks) | | 9176 | | | |  | 9176 | | | |
| Marginal R^2^ / Conditional R^2^ | | 0.013 / 0.411 | | | |  | 0.035 / 0.404 | | | |

**Table C.** Model predicting intra-individual differences in 0-back and 2-back task accuracy from youth cognitive composite network strength in the ABCD Study sample.


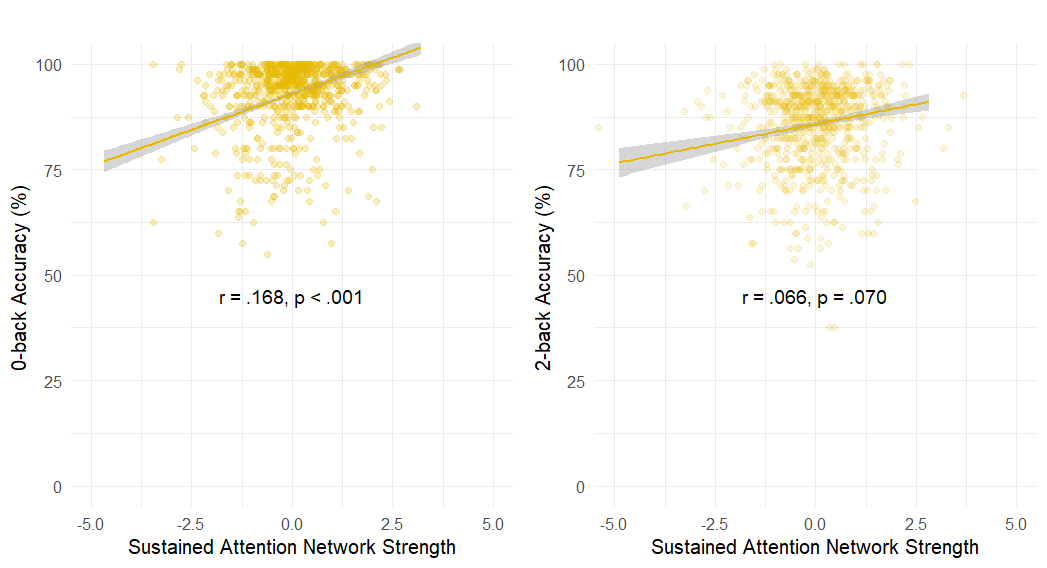


**Fig F.** Relationship between sustained attention network strength and *n*-back accuracy in the adult HCP sample. The data for this figure are available at NDA study 1849 [10.15154/1528288](http://dx.doi.org/10.15154/1528288).

|  | | **0-back Accuracy** | | | | | | | |  | | **2-back Accuracy** | | | | | | |  |
| --- | --- | --- | --- | --- | --- | --- | --- | --- | --- | --- | --- | --- | --- | --- | --- | --- | --- | --- | --- |
| *Predictors* | | *Beta* | | *CI* | | *t* | | *p* | |  | | *Beta* | *CI* | | *t* | | *p* | |  |
| *(Intercept)* | | 0.07 | | [-0.09, 0.22] | | 0.86 | | 0.390 | |  | | 0.20 | [0.04, 0.35] | | 2.44 | | **0.015** | |  |
| *Age:* | |  | |  | |  | |  | |  | |  |  | |  | |  | |  |
|  | *Age 26-30* | | 0.10 | | [-0.09, 0.28] | | 1.03 | | 0.304 | |  | 0.03 | | [-0.16, 0.21] | | 0.30 | | 0.768 | |
|  | *Age 31-35* | | -0.16 | | [-0.35, 0.04] | | -1.59 | | 0.113 | |  | -0.21 | | [-0.41, -0.02] | | -2.14 | | **0.033** | |
|  | *Age 36+* | | 0.02 | | [-0.60, 0.65 | | 0.07 | | 0.941 | |  | -0.31 | | [-0.75, 0.50] | | -0.40 | | 0.691 | |
| *Female* | | -0.11 | | [-0.25, 0.04] | | -1.46 | | 0.144 | |  | | -0.25 | [-0.40, -0.11] | | -3.41 | | **0.001** | |  |
| *Motion* | | -0.08 | | [-0.15, -0.01] | | -2.29 | | **0.022** | |  | | -0.09 | [-0.16, -0.02] | | -2.51 | | **0.012** | |  |
| Sustained Attention | | 0.16 | | [0.09, 0.23] | | 4.51 | | **<0.001** | |  | | 0.07 | [-0.00, 0.14] | | 1.95 | | 0.051 | |  |
| Observations | | 754 | | | | | | | |  | | 754 | | | | | | |  |
| Marginal R^2^ / Conditional R^2^ | | 0.053 / 0.046 | | | | | | | |  | | 0.049 / 0.041 | | | | | | |  |

**Table D.** Model predicting individual differences in 0-back and 2-back task accuracy from sustained attention network strength in the HCP sample.

|  | | **0-back Accuracy** | | | |  | **2-back Accuracy** | | | |
| --- | --- | --- | --- | --- | --- | --- | --- | --- | --- | --- |
| *Predictors* | | *Beta* | *CI* | *t* | *p* |  | *Beta* | *CI* | *t* | *p* |
| *(Intercept)* | | 0.12 | [-0.02, 0.25] | 1.73 | 0.083 |  | -0.01 | [-0.13, 0.12] | -0.11 | 0.912 |
| *Block Motion* | | -1.83 | [-2.70, -0.96] | -9.26 | **<0.001** |  | -1.41 | [-2.20, -0.63] | -3.53 | **<0.001** |
| *Block Type:* | |  |  |  |  |  |  |  |  |  |
|  | *Face* | 0.38 | [0.32, 0.45] | -12.29 | **<0.001** |  | 0.68 | [0.62, 0.73] | 23.76 | **<0.001** |
|  | *Place* | 0.31 | [-0.25, 0.37] | 9.83 | **<0.001** |  | 0.64 | [0.58, 0.69] | 22.62 | **<0.001** |
|  | *Tools* | 0.22 | [0.16, 0.28] | 6.98 | **<0.001** |  | 0.47 | [0.41, 0.52] | -16.34 | **<0.001** |
| *Run 2 – Run 1* | | 0.21 | [0.17, 0.26] | 9.26 | **<0.001** |  | 0.49 | [0.45, 0.53] | 23.29 | **<0.001** |
| Sustained Attention | | 0.08 | [0.06, 0.11] | 6.36 | **<0.001** |  | 0.01 | [-0.02, 0.03] | 0.59 | 0.558 |
|  | |  |  |  |  |  |  |  |  |  |
| **Random Effects**  **(Subject)** | |  |  |  |  |  |  |  |  |  |
|  | ICC | 0.31_subs_ | | | |  | 0.37_subs_ | | | |
| Observations (blocks) | | 5412 | | | |  | 5412 | | | |
| Marginal R^2^ / Conditional R^2^ | | 0.046 / 0.344 | | | |  | 0.135 / 0.456 | | | |

**Table E.** Model predicting intra-individual differences in 0-back and 2-back task accuracy from sustained attention network strength and block type in the HCP sample.


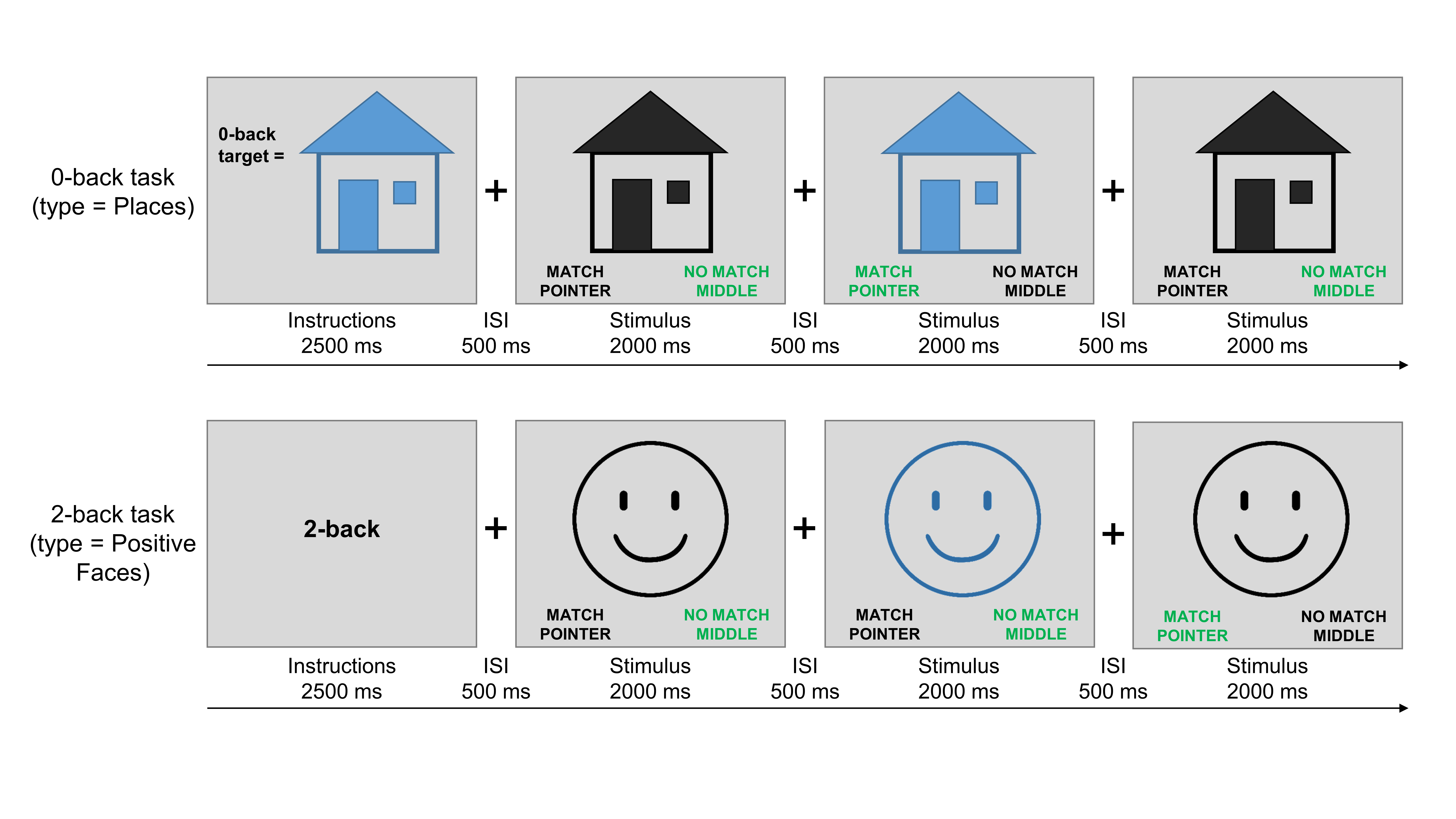


**Fig G.** The instruction and first 3 trials in a block of 0-back task (top, example from Places block type) and 2-back task (bottom, example from Positive Faces block type) are shown in this figure. The correct choice for each trial is indicated in green text. Real stimuli are replaced by schematics for copy right purposes. To see an n-back task used in the ABCD study with example real stimuli please see Casey et al., 2018 [1].

|  | | **0-back Accuracy** | | | |  | **2-back Accuracy** | | | |
| --- | --- | --- | --- | --- | --- | --- | --- | --- | --- | --- |
| *Predictors* | | *Beta* | *CI* | *t* | *p* |  | *Beta* | *CI* | *t* | *p* |
| *(Intercept)* | | -0.00 | [-0.14, 0.13] | -0.03 | 0.978 |  | 0.10 | [-0.01, 0.21] | 1.76 | 0.091 |
| *Age* | | 0.13 | [0.08, 0.18] | 5.23 | **<0.001** |  | 0.14 | [0.09, 0.19] | 5.55 | **<0.001** |
| *Female* | | -0.06 | [-0.16, 0.04] | -1.19 | 0.236 |  | -0.22 | [-0.32, -0.12] | -4.38 | **<0.001** |
| *Motion* | | -0.03 | [-0.08, 0.02] | -1.03 | 0.304 |  | -0.13 | [-0.18, -0.08] | -5.14 | **<0.001** |
| Sustained Attention | | 0.15 | [0.10, 0.20] | 6.10 | **<0.001** |  | -0.04 | [-0.09, 0.01] | -1.72 | 0.086 |
| Working Memory | | 0.11 | [0.06, 0.16] | 4.13 | **<0.001** |  | 0.10 | [0.05, 0.15] | 3.85 | **<0.001** |
|  | |  |  |  |  |  |  |  |  |  |
| **Random Effects**  **(Site)** | |  |  |  |  |  |  |  |  |  |
|  | ICC | 0.06_site_ | | | |  | 0.03_site_ | | | |
| Observations | | 1504 | | | |  | 1545 | | | |
| Marginal R^2^ / Conditional R^2^ | | 0.067 / 0.119 | | | |  | 0.070 / 0.099 | | | |

**Table F.** Model predicting inter-individual differences in 0-back and 2-back task accuracy from sustained attention and working memory network strength values in the ABCD sample with only one of each pair of family members retained randomly (*n* = 1504). Compare these values to **Table 1** in the *Results* section.

|  | | **0-back Accuracy** | | | | |  | **2-back Accuracy** | | | |
| --- | --- | --- | --- | --- | --- | --- | --- | --- | --- | --- | --- |
| *Predictors* | | *Beta* | *CI* | | *t* | *p* |  | *Beta* | *CI* | *t* | *p* |
| *(Intercept)* | | -0.01 | | [-0.13, 0.12] | -0.09 | 0.933 |  | 0.10 | [0.01, 0.19] | 2.11 | **0.044** |
| *Age* | | 0.12 | | [0.07, 0.17] | 4.69 | **<0.001** |  | 0.11 | [0.06, 0.16] | 4.54 | **<0.001** |
| *Female* | | -0.04 | | [-0.14, 0.06] | -0.85 | 0.396 |  | -0.20 | [-0.30, -0.11] | -4.15 | **<0.001** |
| *Motion* | | -0.02 | | [-0.07, 0.03] | -0.80 | 0.422 |  | -0.12 | [-0.17, -0.07] | -4.71 | **<0.001** |
| Cognitive Composite | | 0.19 | | [0.14, 0.24] | 7.20 | **<0.001** |  | 0.26 | [0.20, 0.31] | 9.82 | **<0.001** |
| Sustained attention | | 0.18 | | [0.13, 0.23] | 7.20 | **<0.001** |  | 0.01 | [-0.04, 0.06] | 0.29 | 0.770 |
|  | |  | |  |  |  |  |  |  |  |  |
| **Random Effects**  **(Site)** | |  | |  |  |  |  |  |  |  |  |
|  | ICC | 0.05_site_ | | | | |  | 0.02_site_ | | | |
| Observations | | 1504 | | | | |  | 1504 | | | |
| Marginal R^2^ / Conditional R^2^ | | 0.090 / 0.131 | | | | |  | 0.121 / 0.135 | | | |

**Table G.** Model predicting inter-individual differences in 0-back and 2-back task accuracy from sustained attention and youth cognitive composite network strengths in the ABCD sample with only one of each pair of family members retained randomly (*n* = 1504). Compare these to **Table D in S1 Text**.

|  | | **Recognition Memory d’** | | | |
| --- | --- | --- | --- | --- | --- |
| *Predictors* | | *Beta* | *CI* | *t* | *p* |
| *(Intercept)* | | 0.74 | [0.67, 0.82] | 20.31 | **<0.001** |
| *Age* | | 0.07 | [0.04, 0.10] | 4.27 | **<0.001** |
| *Female* | | -0.03 | [-0.09, 0.04] | -0.80 | 0.422 |
| *Motion* | | 0.00 | [-0.03, 0.04] | 0.21 | 0.832 |
| *n*-back Accuracy | | 0.15 | [0.11, 0.18] | 8.70 | **<0.001** |
| Working Memory | | 0.04 | [0.00, 0.07] | 2.26 | **0.024** |
| Sustained Attention | | -0.02 | [-0.05, 0.01] | -1.31 | 0.192 |
|  | |  |  |  |  |
| **Random Effects**  **(Site)** | |  |  |  |  |
|  | ICC | 0.03_site_ | | | |
| Observations  (subjects) | | 1451 | | | |
| Marginal R^2^ / Conditional R^2^ | | 0.082 / 0.113 | | | |

**Table H.** Model predicting out-of-scanner item memory recognition from sustained attention and working memory network strengths in the ABCD sample with only one of each pair of family members retained randomly. Compare these to **Table 3**.

**Supplementary analysis: Defining *n*-back blocks with a temporal lag**. In each of the two *n*-back runs, there were 8 blocks of 0- and 2-back tasks (4 of each, one per stimulus category). All of the main analyses shown in the *Results* use block-wise functional connectivity matrices for which block start and end times were the first stimulus onset and last stimulus offset times for trials in the block, respectively. However, the rest period between blocks were not equally distributed for all 8 blocks in a run, and instead followed the design shown in the **Fig H in S1 Text**. As shown in the figure, there was enough time before the odd blocks (i.e., blocks 1, 3, 5, and 7) for the BOLD response from the previous *n*-back block to return to baseline, but this was not the case for the even blocks (i.e., blocks 2, 4, 6, 8). As such, in a post hoc analysis we calculated functional connectivity matrices for the even blocks with a 6-s shift (7 or 8 TRs based on rounding the exact timings for each participant) to allow for further separation of the BOLD signal from these blocks with their prior blocks. This is shown in **Fig H in S1 Text** red intervals (compared to blue intervals in the analyses presented in the *Results* sections). We then repeated all ABCD analyses using these shifted functional connectivity matrices. Results were similar to those reported in the non-shifted analysis, except for the **Study 1.3** results shown in **Table 3**. Therefore, we report those results with the time-shifted functional connectivity matrices here. With the time-shifted matrices, working memory network strength still predicted subsequent recognition memory for *n*-back stimuli, although the strength of the association was weaker (*r* = .093, *p* < .001 vs. *r* = .123 in original analysis) and was no longer significant above and beyond in-scanner *n*-back performance (shown in **Table H in S1 Text**).


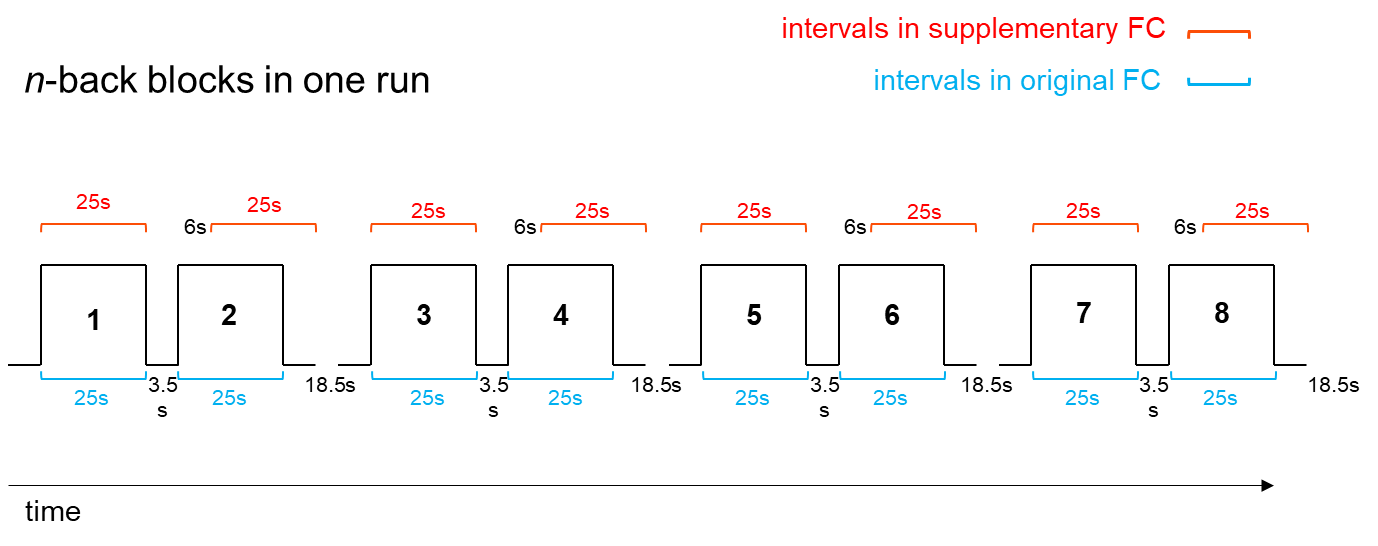


**Fig H.** The time-course of *n*-back blocks (from the beginning of the first trial’s stimulus onset to the end of the last trial’s stimulus offset time in the block) in a run in the ABCD n-back task. While there is only ~3.5-sec interval prior to start of even blocks, there is an additional 15-sec rest interval after every other block of *n*-back.

|  | | **Recognition Memory d’** | | | |
| --- | --- | --- | --- | --- | --- |
| *Predictors* | | *Beta* | *CI* | *t* | *p* |
| *(Intercept)* | | 0.73 | [0.66, 0.79] | 23.22 | **<0.001** |
| *Age* | | 0.06 | [0.03, 0.09] | 3.97 | **<0.001** |
| *Female* | | -0.02 | [-0.08, 0.04] | -0.66 | 0.511 |
| *Motion* | | 0.01 | [-0.02, 0.04] | 0.73 | 0.468 |
| *n*-back Accuracy | | 0.18 | [0.15, 0.21] | 10.85 | **<0.001** |
| Working Memory | | 0.03 | [-0.01, 0.06] | 1.59 | 0.113 |
| Sustained Attention | | -0.02 | [-0.05, 0.01] | -1.34 | 0.179 |
|  | |  |  |  |  |
| **Random Effects**  **(Site)** | |  |  |  |  |
|  | ICC | 0.02_site_ | | | |
| Observations  (subjects) | | 1489 | | | |
| Marginal R^2^ / Conditional R^2^ | | 0.102 / 0.118 | | | |

**Table I.** Working memory network strength measured in the 6-sec shifted manner described above is not significantly related to subsequent recognition memory for *n*-back task stimuli after adjusting for nuisance variables and *n*-back performance itself (compare to **Table 3**).

***Supplementary results with less strict head-motion exclusion criteria*** ***in the ABCD Study sample:***

In addition to the participation weight analysis, we also re-analyzed the data with a less stringent motion threshold of FD mean < .5 mm and FD max < 5 mm to retain more of the ABCD Study sample. The sample size for this FD threshold was n = 3225, which is double the sample with the more stringent FD threshold in the main analyses (FD mean < .2 mm and FD max < 2 mm; n = 1545). Below are the results of the analyses with the .5 mm threshold. The results showed that two of the betas were modestly larger in the larger sample (*p* = .025 and *p* = .054), and the other comparisons show non-significant changes (*p*s > .05) (see below). Since no significant decreases were found, these results echo the same findings from the more restricted sample.

1) Individual differences in sustained attention and working memory network strength are differentially related to individual differences in children’s 0-back (left) and 2-back (right) performance, respectively. Note: Comparing to **Table 1** in the main text with n = 1545 shows no significant changes in the beta coefficient for SA (0-back: *p* = .847, 2-back: *p* = .383 based on Bayesian posterior probability distributions for the betas). The beta coefficient for WM network strength predicting 0-back accuracy is also unchanged (*p* = .565) and it is slightly increased for 2-back accuracy (*p* = .025).

|  | | **0-back Accuracy** | | | |  | **2-back Accuracy** | | | |
| --- | --- | --- | --- | --- | --- | --- | --- | --- | --- | --- |
| *Predictors* | | *Beta* | *CI* | *t* | *p* |  | *Beta* | *CI* | *t* | *p* |
| *(Intercept)* | | 0.03 | [-0.06, 0.12] | 0.68 | 0.499 |  | 0.10 | [0.00, 0.21] | 1.88 | 0.060 |
| *Age* | | 0.13 | [0.10, 0.17] | 7.83 | **<0.001** |  | 0.14 | [0.10, 0.17] | 8.02 | **<0.001** |
| *Female* | | -0.07 | [-0.14, -0.00] | -2.09 | **0.037** |  | -0.21 | [-0.27, -0.14] | -6.29 | **<0.001** |
| *Motion* | | -0.07 | [-0.11, -0.03] | -3.61 | **<0.001** |  | -0.12 | [-0.16, -0.09] | -6.63 | **<0.001** |
| Sustained Attention Network Strength | | 0.14 | [0.10, 0.17] | 7.79 | **<0.001** |  | -0.04 | [-0.07, -0.00] | -2.22 | **0.027** |
| Working Memory Network Strength | | 0.10 | [0.06, 0.16] | 4.19 | **<0.001** |  | 0.14 | [0.11, 0.18] | 7.77 | **<0.001** |
|  | |  |  |  |  |  |  |  |  |  |
| **Random Effects**  **(Site)** | |  |  |  |  |  |  |  |  |  |
|  | ICC | 0.03_site_ | | | |  | 0.04_site_ | | | |
| Observations | | 3225 | | | |  | 3225 | | | |
| Marginal R^2^ / Conditional R^2^ | | 0.076 / 0.103 | | | |  | 0.084 / 0.127 | | | |

**Table J.** Regression of n-back accuracy against network strength values across ABCD participants with liberal head motion criteria.

2) Block-by-block changes in sustained attention and working memory networks strength values are differentially related to within-subject changes in children’s block-wise 0-back and 2-back performance in children, respectively. Note: Comparing to **Table 2** in the main text with n = 1545 shows no significant changes in the beta coefficients for SA (0-back: *p* = .548, 2-back: *p* = .583) and WM network strengths (0-back: *p* = .673, 2-back: *p* = .381) tracking accuracy values.

|  | | **0-back Accuracy** | | | |  | **2-back Accuracy** | | | |
| --- | --- | --- | --- | --- | --- | --- | --- | --- | --- | --- |
| *Predictors* | | *Beta* | *CI* | *t* | *p* |  | *Beta* | *CI* | *t* | *p* |
| *(Intercept)* | | 0.06 | [0.03, 0.09] | 3.86 | **<0.001** |  | 0.02 | [-0.01, 0.05] | 1.20 | 0.232 |
| *Block Motion* | | -0.06 | [-0.08, -0.05] | -9.16 | **<0.001** |  | -0.06 | [-0.08, -0.05] | -9.30 | **<0.001** |
| *Block Type:* | |  |  |  |  |  |  |  |  |  |
|  | *Negative Face* | -0.10 | [-0.13, -0.08] | -7.08 | **<0.001** |  | -0.01 | [-0.04, 0.02] | -0.62 | 0.537 |
|  | *Positive Face* | -0.01 | [-0.04, 0.02] | -0.47 | 0.635 |  | -0.02 | [-0.05, 0.01] | -1.55 | 0.121 |
|  | *Place* | -0.21 | [-0.24, -0.18] | -14.05 | **<0.001** |  | -0.33 | [-0.36, -0.30] | -22.45 | **<0.001** |
| *Run 2 – Run 1* | | 0.03 | [0.01, 0.05] | 2.70 | **0.007** |  | 0.14 | [0.12, 0.16] | 12.40 | **<0.001** |
| Block Sustained Attention Network Strength | | 0.07 | [0.06, 0.08] | 11.20 | **<0.001** |  | -0.01 | [-0.03, -0.00] | -2.51 | **0.012** |
| Block Working Memory Network Strength | | 0.05 | [0.04, 0.06] | 7.74 | **<0.001** |  | 0.04 | [0.03, 0.05] | 6.16 | **<0.001** |
|  | |  |  |  |  |  |  |  |  |  |
| **Random Effects**  **(Subject)** | |  |  |  |  |  |  |  |  |  |
|  | ICC | 0.41_subs_ | | | |  | 0.42_subs_ | | | |
| Observations (blocks) | | 20999 | | | |  | 21000 | | | |
| Marginal R^2^ / Conditional R^2^ | | 0.022 / 0.422 | | | |  | 0.030 / 0.441 | | | |

**Table K.** Regression of n-back accuracy against network strength values in blocks within participants using ABCD participants with liberal head motion criteria.

3) Working memory network strength during in-scanner *n*-back task performance is related to subsequent recognition memory for *n*-back task stimuli after adjusting for nuisance variables and even *n*-back performance itself. Note: Comparing to **Table 3** in the main text with n = 1489 shows no significant change in the beta coefficient for WM network strength predicting recognition memory d’ (*p* = .174).

|  | | **Recognition Memory d’** | | | |
| --- | --- | --- | --- | --- | --- |
| *Predictors* | | *Beta* | *CI* | *t* | *p* |
| *(Intercept)* | | 0.64 | [0.59, 0.69] | 24.44 | **<0.001** |
| *Age* | | 0.04 | [0.02, 0.06] | 3.47 | **0.001** |
| *Female* | | 0.04 | [-0.00, 0.08] | 1.89 | 0.059 |
| *Motion* | | -0.02 | [-0.05, 0.00] | -1.82 | 0.069 |
| *n*-back Accuracy | | 0.20 | [0.17, 0.22] | 17.22 | **<0.001** |
| Working Memory Network Strength | | 0.05 | [0.02, 0.07] | 3.93 | **<0.001** |
| Sustained Attention Network Strength | | -0.04 | [-0.06, -0.02] | -3.43 | **0.001** |
|  | |  |  |  |  |
| **Random Effects**  **(Site)** | |  |  |  |  |
|  | ICC | 0.02_site_ | | | |
| Observations  (subjects) | | 3112 | | | |
| Marginal R^2^ / Conditional R^2^ | | 0.125 / 0.143 | | | |

**Table L.** Regression of recognition memory performance against network strength values across ABCD participants with liberal head motion criteria.

4) Model predicting individual differences in 0-back and 2-back task accuracy from cognitive composite network strength. Note: Comparing to **Table A** in the S1 Text with n = 1545 shows no significant but marginal increases in the beta coefficient for cognitive composite network strength predicting 0-back (*p* = .081) and 2-back (*p* = .054) accuracy values.

|  | | **0-back Accuracy** | | | | |  | **2-back Accuracy** | | | |
| --- | --- | --- | --- | --- | --- | --- | --- | --- | --- | --- | --- |
| *Predictors* | | *Beta* | *CI* | | *t* | *p* |  | *Beta* | *CI* | *t* | *p* |
| *(Intercept)* | | 0.02 | | [-0.06, 0.10] | 0.38 | 0.703 |  | 0.10 | [0.01, 0.19] | 2.30 | **0.021** |
| *Age* | | 0.13 | | [0.10, 0.16] | 7.57 | **<0.001** |  | 0.11 | [0.08, 0.15] | 7.02 | **<0.001** |
| *Female* | | -0.04 | | [-0.11, 0.02] | -1.24 | 0.214 |  | -0.29 | [-0.25, -0.13] | -5.86 | **<0.001** |
| *Motion* | | -0.09 | | [-0.13, -0.06] | -5.26 | **<0.001** |  | -0.11 | [-0.14, -0.07] | -6.40 | **<0.001** |
| Cognitive Composite | | 0.22 | | [0.19, 0.26] | 12.59 | **<0.001** |  | 0.30 | [0.27, 0.33] | 17.59 | **<0.001** |
|  | |  | |  |  |  |  |  |  |  |  |
| **Random Effects**  **(Site)** | |  | |  |  |  |  |  |  |  |  |
|  | ICC | 0.01_site_ | | | | |  | 0.01_site_ | | | |
| Observations | | 3225 | | | | |  | 3225 | | | |
| Marginal R^2^ / Conditional R^2^ | | 0.093 / 0.111 | | | | |  | 0.154 / 0.177 | | | |

**Table M.** Regression of n-back accuracy against cognitive composite network strength values across ABCD participants with liberal head motion criteria.

5) The data-driven latent variables (LV) for the sustained attention (left column) and working memory (right column) PLS regressions with mean FD < .5 mm threshold. Note: Comparing to **Fig 7** in the main text with *n* =1545 (mean FD < .2 mm) for the ABCD Study sample shows consistent patterns and no changes in the interpretation of the results (correlations of network patterns between the two analyses: SA LV1: *r* = .992, LV2: *r* = .748, WM LV1: *r* = .914, LV2: *r* = .882; all *p*s <.001). In other words, the primary LVs are still the FC patterns common between youth and adults sample and the second LVs are patterns that are differentially related to performance between youth and adults. In addition, the first LV is still significantly more dominant for the SA PLS compared to the WM PLS and the 2^nd^ LV is more dominant for the WM PLS than SA PLS (*p* < 1/200 for comparison of σ_XY1_ in SA vs. WM or σ_XY2_ in WM vs. SA) despite the difference becoming weaker compared to the lower-motion analysis.


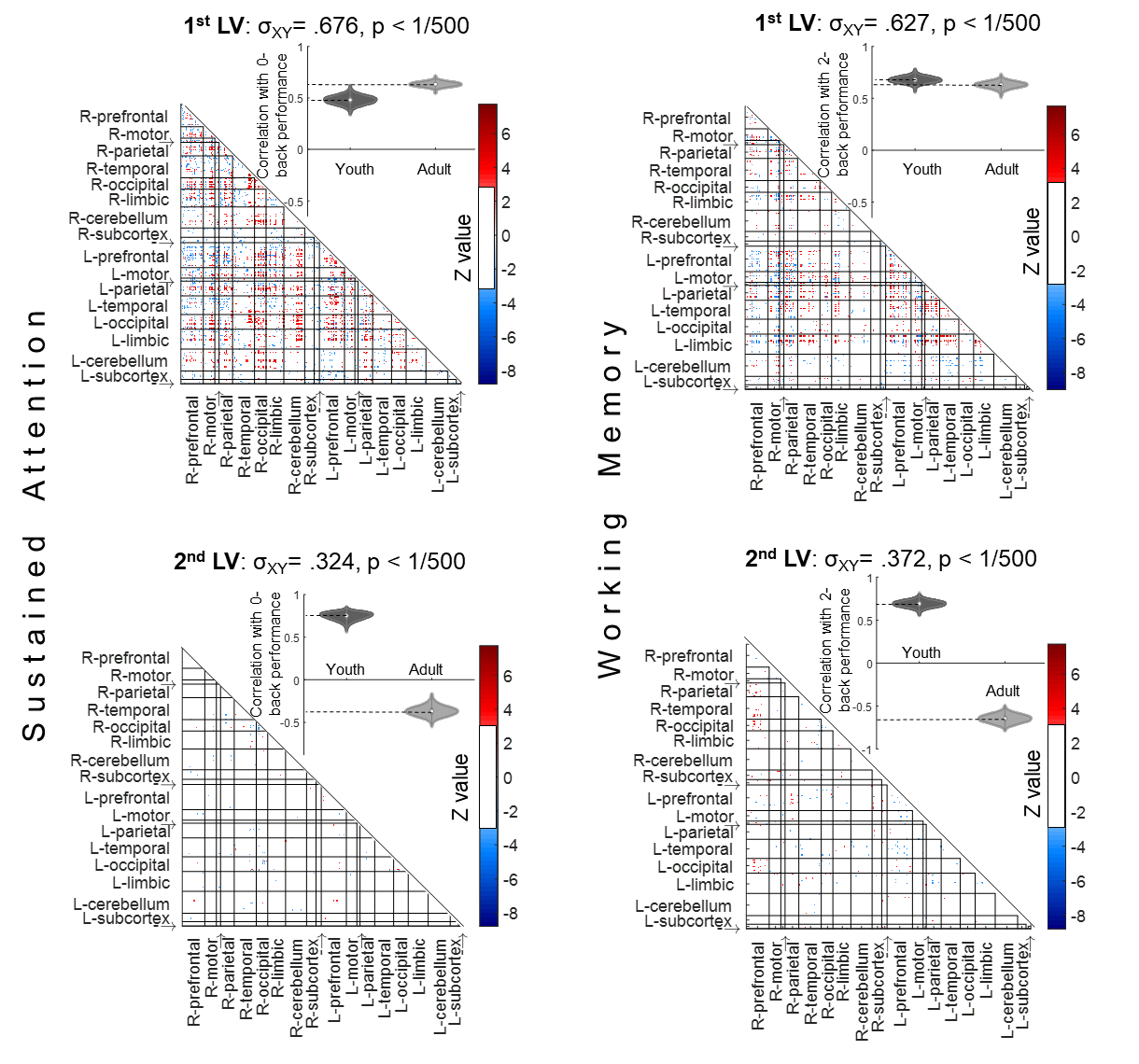


**Fig I.** PLS regression results including ABCD participants with liberal head motion criteria. The data for this figure are available at NDA study 1849 [10.15154/1528288](http://dx.doi.org/10.15154/1528288).

References

1. Casey BJ, Cannonier T, Conley MI, Cohen AO, Barch DM, Heitzeg MM, Soules ME, Teslovich T, Dellarco DV, Garavan H, Orr CA. The adolescent brain cognitive development (ABCD) study: imaging acquisition across 21 sites. Developmental cognitive neuroscience. 2018 Aug 1;32:43-54.
