# Supplementary material for: Differences in the functional brain architecture of sustained attention and working memory in youth and adults
Source: PLoS Biol. 2022 Dec 21;20(12):e3001938. doi: 10.1371/journal.pbio.3001938 (PMC9815648; doi:10.1371/journal.pbio.3001938)
Supplement: Attachment — Submitted filename: Response_to_reviews_PlosBio_Oct22_final.docx [file pbio.3001938.s002.docx]

We appreciate the constructive reviews from the editor and the reviewers. Please see the review notes and responses below. Non-indented text in *italics* = editor & reviewer comments; responses are in blue ink; quoted text from the manuscript are in black ink but highlighted if text was new/changed in the current version of the manuscript

**ACADEMIC EDITOR COMMENTS:**

*I would like the authors to consider the following additional changes/additions:*

*1) Avoid discussing the word "change" in the context of developmental differences.*

**Response E1.** We have now removed the word ‘change’ in the following sentences in the Abstract, Results, and Discussion Sections:

Abstract last sentence: “Thus, functional connectivity predicts SA and WM in youth, with networks predicting WM performance ~~changing more from preadolescence to adulthood~~ differing more between youths and adults than those predicting SA.”

Results last paragraph: “These analyses further support the idea of more differences ~~change~~ between preadolescent and adult networks supporting working memory and more similarity between preadolescent and adult networks supporting sustained attention.”

Discussion:

First paragraph: “Across two studies, we applied different approaches using connectome-based predictions to reveal ~~developmental~~ ~~change~~ differences in the functional architecture of sustained attention and working memory in preadolescence and adulthood.”

Sixth paragraph: “These two comparisons converge to suggest the possibility of developmental ~~changes~~ differences in the functional architecture of working memory ~~during~~ in preadolescence ~~into~~ and adulthood.”

Last paragraph: “Therefore, sustained attention and working memory are overlapping but distinguishable cognitive constructs in the preadolescent brain, with functional connectivity patterns of working memory ~~changing more over adolescence and into adulthood~~ differing more between youth and adults than those of sustained attention.”

*2) Having a supplementary analysis that uses less strict exclusion criteria due to head movements to examine some of the concerns by Reviewer 1. I think that nearly 2/3 exclusion of the sample at this age (9-11) is very rare, and I wonder why is it so high.*

**Response E2.** We appreciate the importance of showing consistent results in a sample with less strict exclusion criteria. We would like to note that the percent of participants included in our final sample, however, is consistent with a recent publication using ABCD Study data from Marek et al. (2022, Nature). Because head motion is pervasive at this age and can bias studies of functional connectivity, we elected to err on the side of caution and exclude high-motion data. We have now included two supplementary analyses to address this point and Reviewer 1’s concern about clinical populations.

First, as suggested by the editor, we performed all ABCD Study data analyses again with frame-wise head displacement (FD) mean < .5 and FD max < 5 mm which increased the sample size by 1680 (compared to our original inclusion threshold of FD mean < .2 and FD max < 2 mm analyses). All analyses, detailed below, produced very similar results to the more stringent motion exclusion analyses (see the highlighted text below for details). We hypothesize that the consistency in results across motion exclusion thresholds arises because we are using previously published predictive models that were originally defined in low-motion data and we include head motion as a covariate in our regression models. We believe that developing predictive models using high-motion ABCD Study data may compromise their generalizability to other datasets.

Second, in our non-participation-weighted analyses (which originally included sex, age, race/ethnicity categories, and family income), we now added the Child Behavior Checklist (CBCL) mean score, which is a proxy for psychopathology. This more comprehensive non-participation analysis confirmed that, in addition to sociodemographic variables, our results were also not sensitive to representations of low/high CBCL scores in included children compared to full ABCD sample as a proxy for psychopathology (see **Response R1.10** for the non-participation analysis with CBCL).

Here is the added supplementary results section:

***Supplementary results with less strict head-motion exclusion criteria in the ABCD Study sample:***

In addition to the participation weight analysis, we also re-analyzed the data with a less stringent motion threshold of FD mean < .5 mm and FD max < 5 mm to retain more of the ABCD Study sample. The sample size for this FD threshold was *n* = 3225, which is double the sample with the more stringent FD threshold in the main analyses (FD mean < .2 mm and FD max < 2 mm; *n* = 1545). Below are the results of the analyses with the .5 mm threshold, which echo the same findings from the more restricted sample.

1) Individual differences in sustained attention and working memory network strength are differentially related to individual differences in children’s 0-back (left) and 2-back (right) performance, respectively. Note: Comparing to **Table 1** in the main text with *n* = 1545 shows no significant changes in the beta coefficients for SA and WM network strengths predicting 0-back and 2-back accuracy values.

|  | | **0-back Accuracy** | | | |  | **2-back Accuracy** | | | |
| --- | --- | --- | --- | --- | --- | --- | --- | --- | --- | --- |
| *Predictors* | | *Beta* | *CI* | *t* | *p* |  | *Beta* | *CI* | *t* | *p* |
| *(Intercept)* | | 0.03 | [-0.06, 0.12] | 0.68 | 0.499 |  | 0.10 | [0.00, 0.21] | 1.88 | 0.060 |
| *Age* | | 0.13 | [0.10, 0.17] | 7.83 | **<0.001** |  | 0.14 | [0.10, 0.17] | 8.02 | **<0.001** |
| *Female* | | -0.07 | [-0.14, -0.00] | -2.09 | **0.037** |  | -0.21 | [-0.27, -0.14] | -6.29 | **<0.001** |
| *Motion* | | -0.07 | [-0.11, -0.03] | -3.61 | **<0.001** |  | -0.12 | [-0.16, -0.09] | -6.63 | **<0.001** |
| Sustained Attention Network Strength | | 0.14 | [0.10, 0.17] | 7.79 | **<0.001** |  | -0.04 | [-0.07, -0.00] | -2.22 | **0.027** |
| Working Memory Network Strength | | 0.10 | [0.06, 0.16] | 4.19 | **<0.001** |  | 0.14 | [0.05, 0.15] | 3.89 | **<0.001** |
|  | |  |  |  |  |  |  |  |  |  |
| **Random Effects**  **(Site)** | |  |  |  |  |  |  |  |  |  |
|  | ICC | 0.03_site_ | | | |  | 0.04_site_ | | | |
| Observations | | 3225 | | | |  | 3225 | | | |
| Marginal R^2^ / Conditional R^2^ | | 0.076 / 0.103 | | | |  | 0.084 / 0.127 | | | |

2) Block-by-block changes in sustained attention and working memory networks strength values are differentially related to within-subject changes in children’s block-wise 0-back and 2-back performance, respectively. Note: Comparing to **Table 2** in the main text with *n* = 1545 shows no significant changes in the beta coefficients for SA and WM network strengths tracking 0-back and 2-back accuracy values.

|  | | **0-back Accuracy** | | | |  | **2-back Accuracy** | | | |
| --- | --- | --- | --- | --- | --- | --- | --- | --- | --- | --- |
| *Predictors* | | *Beta* | *CI* | *t* | *p* |  | *Beta* | *CI* | *t* | *p* |
| *(Intercept)* | | 0.06 | [0.03, 0.09] | 3.86 | **<0.001** |  | 0.02 | [-0.01, 0.05] | 1.20 | 0.232 |
| *Block Motion* | | -0.06 | [-0.08, -0.05] | -9.16 | **<0.001** |  | -0.06 | [-0.08, -0.05] | -9.30 | **<0.001** |
| *Block Type:* | |  |  |  |  |  |  |  |  |  |
|  | *Negative Face* | -0.10 | [-0.13, -0.08] | -7.08 | **<0.001** |  | -0.01 | [-0.04, 0.02] | -0.62 | 0.537 |
|  | *Positive Face* | -0.01 | [-0.04, 0.02] | -0.47 | 0.635 |  | -0.02 | [-0.05, 0.01] | -1.55 | 0.121 |
|  | *Place* | -0.21 | [-0.24, -0.18] | -14.05 | **<0.001** |  | -0.33 | [-0.36, -0.30] | -22.45 | **<0.001** |
| *Run 2 – Run 1* | | 0.03 | [0.01, 0.05] | 2.70 | **0.007** |  | 0.14 | [0.12, 0.16] | 12.40 | **<0.001** |
| Block Sustained Attention Network Strength | | 0.07 | [0.06, 0.08] | 11.20 | **<0.001** |  | -0.01 | [-0.03, -0.00] | -2.51 | **0.012** |
| Block Working Memory Network Strength | | 0.05 | [0.04, 0.06] | 7.74 | **<0.001** |  | 0.04 | [0.03, 0.05] | 6.16 | **<0.001** |
|  | |  |  |  |  |  |  |  |  |  |
| **Random Effects**  **(Subject)** | |  |  |  |  |  |  |  |  |  |
|  | ICC | 0.41_subs_ | | | |  | 0.42_subs_ | | | |
| Observations (blocks) | | 20999 | | | |  | 21000 | | | |
| Marginal R^2^ / Conditional R^2^ | | 0.022 / 0.422 | | | |  | 0.030 / 0.441 | | | |

3) Working memory network strength during in-scanner *n*-back task performance is related to subsequent recognition memory for *n*-back task stimuli after adjusting for nuisance variables and even *n*-back performance itself. Note: Comparing to **Table 3** in the main text with *n* = 1489 shows no significant change in the beta coefficient for WM network strength predicting recognition memory *d’*.

|  | | **Recognition Memory d’** | | | |
| --- | --- | --- | --- | --- | --- |
| *Predictors* | | *Beta* | *CI* | *t* | *p* |
| *(Intercept)* | | 0.64 | [0.59, 0.69] | 24.44 | **<0.001** |
| *Age* | | 0.04 | [0.02, 0.06] | 3.47 | **0.001** |
| *Female* | | 0.04 | [-0.00, 0.08] | 1.89 | 0.059 |
| *Motion* | | -0.02 | [-0.05, 0.00] | -1.82 | 0.069 |
| *n*-back Accuracy | | 0.20 | [0.17, 0.22] | 17.22 | **<0.001** |
| Working Memory Network Strength | | 0.05 | [0.02, 0.07] | 3.93 | **<0.001** |
| Sustained Attention Network Strength | | -0.04 | [-0.06, -0.02] | -3.43 | **0.001** |
|  | |  |  |  |  |
| **Random Effects**  **(Site)** | |  |  |  |  |
|  | ICC | 0.02_site_ | | | |
| Observations  (subjects) | | 3112 | | | |
| Marginal R^2^ / Conditional R^2^ | | 0.125 / 0.143 | | | |

4) Model predicting individual differences in 0-back and 2-back task accuracy from cognitive composite network strength. Note: Comparing to **Table S1** in the supplementary text with *n* = 1545 shows no significant change in the beta coefficient for cognitive composite network strength predicting 0-back and 2-back accuracy values.

|  | | **0-back Accuracy** | | | | |  | **2-back Accuracy** | | | |
| --- | --- | --- | --- | --- | --- | --- | --- | --- | --- | --- | --- |
| *Predictors* | | *Beta* | *CI* | | *t* | *p* |  | *Beta* | *CI* | *t* | *p* |
| *(Intercept)* | | 0.02 | | [-0.06, 0.10] | 0.38 | 0.703 |  | 0.10 | [0.01, 0.19] | 2.30 | **0.021** |
| *Age* | | 0.13 | | [0.10, 0.16] | 7.57 | **<0.001** |  | 0.11 | [0.08, 0.15] | 7.02 | **<0.001** |
| *Female* | | -0.04 | | [-0.11, 0.02] | -1.24 | 0.214 |  | -0.29 | [-0.25, -0.13] | -5.86 | **<0.001** |
| *Motion* | | -0.09 | | [-0.13, -0.06] | -5.26 | **<0.001** |  | -0.11 | [-0.14, -0.07] | -6.40 | **<0.001** |
| Cognitive Composite | | 0.22 | | [0.19, 0.26] | 12.59 | **<0.001** |  | 0.30 | [0.27, 0.33] | 17.59 | **<0.001** |
|  | |  | |  |  |  |  |  |  |  |  |
| **Random Effects**  **(Site)** | |  | |  |  |  |  |  |  |  |  |
|  | ICC | 0.01_site_ | | | | |  | 0.01_site_ | | | |
| Observations | | 3225 | | | | |  | 3225 | | | |
| Marginal R^2^ / Conditional R^2^ | | 0.093 / 0.111 | | | | |  | 0.154 / 0.177 | | | |

5) The data-driven latent variables (LV) for the sustained attention (left column) and working memory (right column) PLS regressions with mean FD < .5 mm threshold. Note: Comparing to **Figure 7** in the main text with *n* =1545 (mean FD < .2 mm) for the ABCD Study sample shows consistent patterns and no changes in the interpretation of the results. In other words, the primary LVs are still the FC patterns common between the youth and adult samples and the second LVs are patterns that are differentially related to performance in youth and adults. In addition, the first LV is still significantly more dominant for the SA PLS compared to the WM PLS and the second LV is more dominant for the WM PLS than SA PLS (*p* < 1/200 for comparison of σ_XY1_ in SA vs. WM or σ_XY2_ in WM vs. SA) despite the difference becoming weaker compared to the lower-motion analysis.

In summary, for our Response E2, analyses 1-5 show that with *n* = 3225 based on a more liberal motion exclusion threshold of 0.5 mm FD, our findings are consistent with the more restricted sample of *n* = 1545 obtained with the more conservative .2 mm FD movement threshold.

*3) Discuss the implications of their findings for cognitive/biological theories of brain development such as Mark H. Johnson's interactive specialisation approach, and Anderson's neural re-use hypothesis.*

**Response E3.** Thank you for pointing us to this work. We have now added the following paragraph in the discussion section:

“When putting these results in the context of broader theories of functional brain development, our findings are consistent with two big picture frameworks. First, our finding that there is dynamicity in the brain-behavior mapping of both sustained attention and working memory in development (though not to the same extent for each process) is consistent with the interactive specialization hypothesis (Johnson, 2011). This framework posits that developmental change in cognitive skills or behavior is accompanied by widespread changes across *network* of regions, rather than maturation of single brain regions (Johnson, 2011). Second, we found that overlapping networks support sustained attention and working memory abilities between preadolescents and adults. This is consistent with the neural reuse hypothesis (Anderson, 2010), which posits that regions of the brain are ‘reused’ throughout development such that each will end up participating in multiple functions and involved in diverse tasks across multiple cognitive domains (Anderson, 2016). Prior empirical evidence supporting these two frameworks has been primarily from earlier stages of life than preadolescence and our approach, combined with other work in the development of brain structure (e.g., Bethlehem et al., 2022) may shine some light on these theories in other stages of lifespan.”

**Reviewer #1:** *This very rigorous manuscript demonstrates that a priori connectomic brain measures used to predict sustained attention and working memory, in adult datasets, generalize to the predict a proxy for sustained attention (0-back performance) and working memory (the same 2-back task) in a group of children from the ABCD dataset. A main strength of this study is that it demonstrates that some aspects of functional brain architecture that support attention and WM in adults are present in children. The second main strength is that it further corroborates the utility and generalizability of these CPMs across datasets and different populations. On the other hand, there are weaknesses in some unwarranted conclusions drawn from these analyses that are overstated or misleading.*

**Response R1.1.** Thank you for your careful assessment and feedback. We have now added new analyses, explanations, and clarifications in the text which we believe have strengthened the manuscript.

1. *From the title, and throughout the manuscript, differences in prediction accuracy across CPMs and datasets are used as evidence for "developmental differences" in sustained attention vs. WM. I do not think these conclusions are warranted, as I do not believe that differences in prediction of CPMs themselves drawn from different datasets (composite, SA, WM) can be easily interpreted.*

**Response R1.2.** This point is well taken. We have edited the title to “Connectome-based predictions reveal differences in the functional architecture of sustained attention and working memory in youth and adults” to describe “difference” rather than change. We have also edited wording throughout the paper, described in **Response E1**, to avoid drawing strong conclusions about developmental change.

At the same time, we would like to emphasize that we use both the comparative fit of CPMs and the data-driven PLS regressions to converge on a conclusion that working memory networks differ more between youth and adults than do sustained attention networks. Below, we show that the alternative hypotheses suggested—that task or dataset differences explain different results for the working memory and sustained attention networks—does not explain the results. Rather, differences in developmental change may best explain the observed pattern of results. However, the new language in and framing of the paper presents this as one possible explanation rather than a definitive conclusion of our results.

*Similarly, ABCD vs. HCP datasets have many differences besides age, including scan parameters, data size, data quality, etc., thus prediction comparisons across datasets are difficult to interpret.*

**Response R1.3.** We agree with the reviewer that the ABCD Study and HCP datasets differ in many respects. We highlight these differences in an expanded limitation paragraph of our Discussion. However, we would like to point out two analyses that suggest that age differences (rather than other differences between the ABCD Study and HCP datasets) drive the primary results of Study 2 of our paper: that sustained attention networks are more similar between youth and adults than working memory networks are.

First, we would like to address differences in scan parameters and data size between the datasets. When we compared sustained attention model fits across participants in the ABCD and the HCP samples (**Manuscript** **Figure 6**) and performed the PLS regressions of SA and WM performance in both datasets together (**Manuscript** **Figure 7**), functional connectivity matrices were z-scored within each participant when used in the respective analyses. So, only scanner differences between HCP and ABCD studies that cause spatially non-homogenous differences between HCP and ABCD scans could interfere with age-related differences. This helps account for scanner differences related to whole-brain mean and variance of the signal in the connectivity matrices as well as scanning parameters related to global signal to noise ratio (e.g., TR and voxel dimensions). Related to data size, in the PLS regression we randomly sampled 754 participants from ABCD across 200 resamples to equate the size of the ABCD and HCP groups in the analysis. The length of the n-back data used in each dataset is very similar (16 blocks of ~25 seconds in both ABCD and HCP studies).

Our PLS analysis further rules out spatially non-homogenous data quality differences as drivers of our primary result that sustained attention networks are more similar between youth and adults than working memory networks. In this analysis (**Manuscript** **Figure 7**), we identified patterns of functional connections that predict behavior in a *group-specific* and *group-general* manner (again using FC matrices z-scored within participants). We found that the first latent variable, which represents connections that are positively related to performance for both youth and adult groups, is stronger for sustained attention than working memory (i.e., the age-general latent variable is more dominant in the PLS regression of SA than WM). Also, the second LV, which represents the group-specific connections, is stronger for WM than SA. When comparing same LVs (e.g., LV1) across the two tasks (0-back and 2-back) the contrast is not between the HCP and the ABCD datasets but rather is between the tasks. This finding that the *age-general* latent variable is significantly less dominant for working memory than it is for sustained attention cannot be driven by a main effect of dataset on data quantity or quality (even a spatially non-homogenous quality difference) because the comparison is between 0-back fMRI data vs 2-back fMRI in both datasets (note analogous argument for LV2 applies). For data quality or quantity to impact our conclusion that SA networks are more similar between youth and adults than WM networks, there would have to be an interaction of data quantity/quality with task type (0-back vs. 2-back), such that data from one task is differentially impacted by the non-age-related factors. Because these task blocks were collected in the same fMRI runs in each respective dataset we believe this is unlikely.

For these reasons, we believe that, while differences in scan parameters and data size and quality between datasets are important factors to consider, they are not the primary drivers of our results.

*To compound it, these statistically significant differences in prediction are notably small in terms of variance explained.*

**Response R1.4.** We agree with the reviewer. Our observed effect sizes are consistent with Marek et al., 2022 <https://www.nature.com/articles/s41586-022-04492-9>, using ABCD, HCP, and UK Biobank datasets where, on average, out-of-sample multivariate brain-behavior associations (mean *r* = 0.17) were smaller than in-sample associations (mean *r* = 0.46). However, it is important to mention that these smaller effect sizes from large samples are reported to be more robust and replicable than larger effect sizes from small samples (Marek et al., 2022). We thus believe that while differences are small in terms of explained variance in predictive power, they are robust, reliable, and theoretically meaningful. We have now also mentioned this in the limitation paragraph.

*Further, the SA CPM was based on a different task (not 0-back) and perhaps it captures similar variance in kids and adults because it doesn't generalize compared to training and testing on the same exact task. In other words, the SA CPM may be non-superior in adults because it is from a different task.*

**Response R1.5.** This is an important point. Interestingly, however, this hypothesis predicts the opposite pattern of results than we observed here. If model generalizability were primarily driven by task generalizability, the sustained attention network model should generalize *worse* to youth (because it was defined on the gradCPT whereas the youth perform the n-back task) and the working memory model should generalize *better* (because it was defined on the same task, i.e., the *n*-back task). Contrary to this prediction, we find that the sustained attention CPM (built from gradCPT) fits the 0-back data in the ABCD dataset (i.e., a different task) better than WM CPM fits the 2-back in ABCD (even though the WM CPM was built from a similar n-back task).

To further investigate the question of whether task similarity is a primary driver of model generalizability, we trained a CPM to predict 0-back performance in the HCP sample. This CPM is, therefore, trained on the same sample as the WM CPM we used from Avery et al, 2020. We then replaced the Rosenberg et al. 2016 sustained attention CPM (which is trained on adults performing the gradCPT) with this HCP-0-back CPM and applied it to the ABCD Study data.

Consistent with our findings using the gradCPT sustained attention CPM, the HCP 0-back CPM predicts ABCD 0-back accuracy (r_adj_ = .25, p <.001) but not 2-back accuracy (r_adj_ = .05, p =.056). Furthermore, it does so significantly better than the HCP 2-back CPM (from Avery et al.) predicts 2-back accuracy in youth (r_adj_ = .11; p <1/1000 when comparing 0-back CPM’s fit to youth 0-back accuracy and 2-back CPM’s fit to youth 2-back accuracy; p-value calculated based on bootstrapped distribution of r_adj_ between the two models; adjustment in *r* done for age, sex, head motion, and site). This shows that whether we use the Rosenberg et al. 2016 gradCPT CPM or the HCP 0-back SA CPM we will arrive at the same conclusion that adult network predictors of sustained attention generalize to preadolescents’ sustained attention performance, and with better fit than adult network predictors of working memory do on adolescent working-memory data.

We appreciate the opportunity to clarify this point with a new analysis, and have added the results with new HCP 0-back CPM in the manuscript:

“**2.1.3.  *Alternative adult-defined connectivity-based predictor of sustained attention.*** To further assess the finding that there are more differences in the functional architecture of working memory vs. sustained attention from preadolescence to adulthood, we trained a new connectivity-based predictive model for 0-back performance in adults from the HCP dataset’s *n*-back fMRI data in a post hoc analysis. We then applied this HCP-based network predictor of sustained attention instead of the Rosenberg et al. (2016) sustained attention model (which is trained on adults performing a gradual-onset continuous performance task) to ABCD Study data. Consistent with our findings using the Rosenberg et al. (2016) sustained attention model, the HCP-based network predictor of 0-back in adults predicts 0-back accuracy in the youth sample (*r_adj_* = .25, p <.001) but does not predict 2-back performance (*r_adj_* = .05, p =.056). Importantly, we found that this alternative adult sustained attention model fits the 0-back performance of the youths significantly better than the adult working memory model predicts 2-back accuracy in youth (*r_adj_* = .11; p <1/1000 when comparing fits based on bootstrapped distribution of *r_adj_* between the two models). This analysis further supports our finding that networks supporting sustained attention are more consistent between youths and adults compared to those supporting working memory.

*Therefore, I do not believe the results support differential development of neural architecture for SA vs. WM.*

**Response R1.6.** For the reasons provided above we think, with our changes in text and more highlighted nuances and analyses, there is support for this via converging evidence, though within the stated limitations of the study.

*2. While the generalization of these CPMs is exciting, the interpretation of the anatomy is potentially overstated. In the group's prior PNAS work, edge-overlap between models was modest. As such, while these edges are sufficient to predict SA and WM, they may not be necessary. A more rigorous lesion approach would remove these edges, and determine if an alternative CPM with orthogonal edges could predict. This would help justify anatomical specificity.*

**Response R1.7.** Thank you for this important point. We agree that it is generally important to test the anatomical specificity of the predictive networks (but also see our **Response R1.9**). However, we do not see a fair way to implement the specific analysis suggested by the reviewer. Lesioning the sustained attention and working memory CPM edges in the original Rosenberg et al. (2016) and HCP datasets in which these models were defined would leave no edges significantly correlated with behavior to select for new models. We could lesion these edges in the ABCD Study dataset itself and build new models predicting 0-back and 2-back accuracy using cross-validation. However the resulting estimates of predictive power achieved with this internal validation approach would not be comparable to those achieved with our current external (cross-dataset) validation tests, as within-dataset prediction estimates are inflated relative to cross-dataset prediction estimates.

We instead performed two related analyses to test anatomical specificity. We compared the performance of the sustained attention and working memory networks in predicting 0-back and 2-back accuracy in the ABCD Study sample to that of *(a)* canonical networks and *(b)* random sets of edges of similar size in the final analysis in Study 1.1.2. These analyses revealed that the sustained attention and working memory networks significantly outperformed all canonical functional networks (the medial-prefrontal, frontoparietal, default, subcortical-cerebellar, motor, visual I, visual II, and visual-association networks) or random sets of size-matched edges when predicting 0-back and 2-back accuracy, respectively.

These results point to some anatomical specificity of the data-driven CPMs. However, it is almost certainly not the case that the edges in the adult sustained attention CPM, for example, comprise the “end all be all” attention network. Rather, it is a preregistered set of edges that robustly predicts sustained attention function. Future work examining the overlap of edges that predict different measures of sustained attention and working memory in different datasets can help refine the models to the minimum set of edges that still generalizes across datasets. We make progress toward this goal here by making the 0-back HCP-based network predictor and reporting its overlap with the gradCPT SA network, as well as by running the PLSR analysis and making these masks available to other researchers. We have now added this statement in the limitations paragraph to avoid overstating of the functional anatomy of the networks.

*In a related point, more traditional functional network-based parcellations (rather than these more strictly anatomical "networks") would help strengthen the argument that network-based prediction failed to adequately capture behavior, and this could be included in a supplement.*

**Response R1.8.** We appreciate the opportunity to clarify that these networks are functionally defined. Nodes were functionally defined with the 268-node Shen atlas generated by performing clustering on voxel-wise time series of resting state data ([Shen et al., 2013, *NeuroImage*](https://www.sciencedirect.com/science/article/abs/pii/S1053811913005818)). In addition, we compared the predictive power of our data-driven CPMs to that of functionally defined canonical networks (e.g., DMN, frontoparietal, etc.) in the last paragraph of section 1.1.2., *Neuromarkers differentially predict sustained attention and working memory abilities*.

When showing the anatomy of the CPMs in the figures we use the structural labels for color-grouping the locations of parcels involved in edges (e.g., left prefrontal, right temporal, etc.) for visualization only. The relevant section of the manuscript is included here for convenience:

“Additionally, we compared the performance of the sustained attention and working memory models to that of 8 canonical functional networks (the medial-prefrontal, frontoparietal, default, subcortical-cerebellar, motor, visual I, visual II, and visual-association networks; Finn et al., 2015). To do so, we correlated the average strength of all within-network connections in each network with n-back accuracy across participants. The sustained attention and working memory networks significantly outperformed all canonical networks when predicting 0-back and 2-back accuracy, respectively (Z-test between beta coefficients: Zs > 4.74, ps < .001 for the sustained attention network and 0-back accuracy, Zs > 2.39, p values < .017 for the working memory network and 2-back accuracy). Furthermore, the sustained attention and working memory networks also significantly outperform randomly selected size-matched sets of connections from outside these networks for predicting 0-back and 2-back accuracy, respectively (p values < 1/201 for both networks when compared to 200 random networks).”

*Along these lines, if the composite CPM can predict WM and SA, then it further suggests that many different sets of connections can predict cognition broadly and there is not specificity to the WM or SA CPMs per se. How well the weights/edges reproduce is key to interpretation of the anatomical specificity of CPMs.*

**Response R1.9.**

Since the composite CPM is brought up here to refer to a more general concern over specificity of CPMs, we would like to respond first to the general point in continuation to our Response R1.7, but also specifically discuss the composite CPM as well.

In continuation to our Response R1.7, we would like to clarify that the exact anatomy of the functional network predictors is not the main goal of the paper or central to our approach, rather the summary of the multivariate patterns of connectivity (i.e., network strength) is studied. Moreover, even the summary network strengths within the predefined SA and WM networks are not posited as the sole analysis that would justify our conclusions, but newly defined CPMs as well as whole-connectome PLS models are used to compare the SA and WM networks in children vs. adults across different connectome predictors of SA and WM. For example, when we substituted the SA CPM (defined with the gradCPT from Rosenberg et al., 2016) with a new 0-back CPM defined in the HCP dataset our conclusions remained the same. Another example is in section 1.1.1 where the anatomy of SA and WM networks are compared. There we clearly state that the lack of significant overlap between networks is not a sufficient indicator of separation between the two processes, and thus we additionally compare the aggregated network strength measures from the two networks. A final example is from Study 2.2, where we make it clear that the whole-connectome PLS analysis is needed to look at the differences in SA and WM without regards for the functional anatomy of the predefined CPMs (since restricting everything to within the predefined networks would leave the other connections that may well be relevant in a blind spot).

However to be explicit, we have now added this to the limitations paragraph, copied here for convenience:

“There are some limitations to this work. First, we rely on 0-back and 2-back performance to index sustained attention and working memory rather than more traditional tasks like a CPT and visual change detection tasks, as these more traditional paradigms are not included in the ABCD Study. Future work characterizing the generalizability of connectivity-based models to other tasks of attention and working memory can further inform their predictive boundaries. Additionally, work suggests that models can be generalizable and robust with features that vary (e.g., Tian & Zalesky, 2021). It is not the case that the edges in the sustained attention and working memory networks used here are the “end all be all” attention and working memory networks. Rather, they are a preregistered set of edges that robustly predict sustained attention and working memory function. Future work examining the overlap of edges that predict different measures of sustained attention and working memory in different datasets can help to refine the models to the minimum set of edges that still generalizes across datasets. We make progress towards this goal by making the 0-back HCP-based network predictor brain mask and the PLSR analysis brain mask available to other researchers. Second, the within-participant fluctuation effects in Study 1.2 are statistically significant yet modest, and the across-participant effects in Studies 1.1 and 1.3 have effect sizes that are small to medium. This is consistent with a recent report using the ABCD Study data (Marek et al., 2022), where, on average, out-of-sample multivariate brain-behavior associations (mean *r* = 0.17) were smaller than in-sample associations (mean *r* = 0.46). However, it is important to mention that these smaller effect sizes from large samples are reported to be more robust and replicable than larger effects sizes from small samples (Marek et al., 2022). Although the current approach demonstrates the statistical significance and theoretical implications of conservative external model validation analyses, further work is needed to determine the practical significance and potential translational utility of these and other brain-based predictive models. Third, and as mentioned before, in Study 2.2, since the ABCD and HCP samples are not the same cohort, it is difficult to tease apart the age-related versus scanning-related differences between the two samples in the multivariate PLS regression. However, it should be mentioned that the *relative* comparison of the primary LVs’ dominance in sustained attention versus working memory PLS regressions would not be sensitive to the non-age-related factors unless these non-age-related factors *interact* differentially with fMRI data during 0-back versus 2-back blocks.”

We would also like to take the opportunity to clarify the aim of the cognitive composite model analysis. The goal was to build a model in the ABCD Study itself as a “ceiling measure” of predictive power for this dataset. In doing so, we elected to predict cognitive composite scores rather than 0-back and 2-back accuracy for two reasons. First, the cognitive composite measure was calculated from out-of-scanner tasks. Predicting in-scanner task performance could inflate estimates of predictive power within the dataset. Second, the cognitive composite scores are equally correlated with 0-back and 2-back performance scores. So, a cognitive composite model is not biased at the outset to better predict either measure.

After building this model we asked three questions:

1) Does the cognitive composite CPM predict youth 0-back performance better than the adult-defined SA CPM?

2) Does the cognitive composite CPM predict youth 2-back performance better than the adult-defined WM CPM?

3) If its fits are superior to adult CPMs, is the degree of superiority equal for WM and SA?

Results revealed that the cognitive composite CPM does perform better than the adult-defined WM CPM but does *not* perform better than the adult-defined SA CPM. This result, together with the other two analyses from Study 2, provides converging evidence that the functional networks supporting WM in youth are less adult-like than those supporting SA.

3. *Given these first two limitations, while the results do support some common neural architecture for SA and WM in children and adults, I don't believe this is a novel premise, as children activate overlapping regions as adults during cognition, and have at least similar functional network properties. Of course, I still applaud the novelty of the current study's methods, scope, and rigor.*

**Response R1.10.** We understand the issues raised and hope our additional analyses have addressed them. We have also added more theoretical context as to the implications of our findings as suggested by the editor (See **Response E.3**). We would also like to highlight the novelty of our Study 1 results—that adult-defined neuromarkers generalize to an independent sample to predict inter- and intra-individual differences in sustained attention and working memory in youth. That is a novel finding. Second, we very respectfully disagree that our Study 2 findings are not novel. For example, the Study 2 findings are questioned in point #4 below and are described by Reviewer 2 as a “substantial contribution”.

4. *Stepping back, even this conclusion that children use similar brain connections as adults has significant limitations. The sizable data exclusion necessary (>2/3) raises concerns about the generalizability to "normal" or most children for that matter. Excluding the large majority of the ABCD sample for too much movement or task performance leads to the real possibility that this study may be about the highest functioning children, and the most "adult-like" in the sample. This limits the study's generalizability to average children, as well as potentially the clinical utility of these findings (e.g., to ADHD).*

**Response R1.11.** We agree that it is very important to test the generalizability of our conclusions to a larger subset of the ABCD Study sample given that the low-motion sample may not be representative of the full dataset or of children more generally. We performed two types of analyses to address this issue.

First, to correct for any biases due to not being included in the analysis relative to the demographic characteristics of the overall ABCD Study sample, we conducted sensitivity analyses to confirm if the relationships in our between-subject analyses (*n* = 1545) hold with non-participation (non-inclusion) weights (overall ABCD Study sample *n* = 11875). Specifically, results were re-assessed with membership in the analyses weighted by estimated non-participation weights calculated for sex, age, race/ethnicity categories, Child Behavior Checklist (CBCL) mean score (square root transformed), and family income and then combined with the American Census Study (ACS) to ABCD raked propensity scores. These analyses show that the results reported in the low-head-motion sample replicate (some are even slightly strengthened) when the non-participation weights are applied to correct the associations to be representative of the full ABCD sample and the US national census. Our original non-participation weighted analyses included only age, sex, race/ethnicity, family income, and parental education as criteria of representativeness. To address the clinical utility concerns of the reviewer, we added the Child Behavior Checklist (CBCL) mean scores in the non-participation weights in addition to the socioeconomic and demographic factors. After this addition all correlations in Study 1 remained robust to the non-participation weighted corrections. We describe these results in a new section accompanying Manuscript Figure 4:

“Finally, we adjusted for data exclusions on household income, age, maternal education, race/ethnicity, sex, household size, and mean Child Behavior Checklist scores using non-participation sensitivity analysis (see *Methods*). The corrected correlation between working memory network strength and 2-back and 0-back accuracy (r_corrected_ = .134 and r_corrected_ = .115, respectively; ps < .001), as well as between sustained attention network strength and 0-back accuracy (r_corrected_ = .246, p <.001) were significant. Thus, these correlations are robust to the application of post-stratification weights (to account for differences between the full ABCD sample and nationally representative socio-demographics) and non-participation weights (to account for differences between the full ABCD sample and the included sample of 1545 in our analysis with respect to representation of socio-demographics and psychopathology).”

Second, as requested by the reviewing editor, we included a supplementary analysis where the FD threshold for exclusion was set at .5 mm, resulting in a doubled sample size. The addition of these 1680 higher motion participants did not change our findings and conclusions and are elaborated in **Response E.2** (i.e., the results are the same when these additional participants are included).

**Other comments:**

5. *Given skewed performance distribution particularly in the 0-back, the authors should consider reporting Spearman rho or other non-parametric tests, especially given the relatively small parametric effect sizes.*

**Response R1.12.** We have now added the Spearman ρ in the results where Pearson correlations were reported.

6. *Why not consider a 0-back CPM derived from the HCP data to compare more directly across the same tasks?*

**Response R1.13.** We thank the reviewer for this suggestion and have added results based on this new CPM as elaborated in **Response R1.5.**

7. *It would be interesting to consider generalizability across block types when predicting individual differences in study 1.1.*

**Response R1.14.** This is interesting. We include block types as covariates in the within-subject regressions and in our supplementary section “Stimulus type does not explain within-subject relationships between network strength and behavior.” We find that SA and WM network strength does not vary across block types within participants.

In addition, we performed an across-subject analysis similar to Study 1.1 but with *n*-back data broken down by block types (Figure R1.14). The pattern of results is similar to the aggregate analyses. Sustained attention network strength predicts 0-back accuracy across participants using connectivity and accuracy within each block type (Neutral Face, Positive Face, Negative Face, and Place), but not 2-back accuracy. Working memory network strength predicts both 0-back and 2-back accuracy values. The correlations are overall slightly weaker than the aggregate analyses as expected because less data were used to calculate functional connectivity measures (~50 seconds) and behavioral scores (20 trials) in each participant. We believe that in the current manuscript this analysis may become burdensome for readers given the large number of analyses in the text, so it is not included in the supplementary results. However, we are happy to include it at the request of the reviewer or editor.

**Figure R1.14.** Across-participant prediction of 0-back and 2-back accuracy in ABCD using the network models of sustained attention and working memory, where each plot only includes fMRI and behavioral trials within the specified block type (from top to bottom: neutral, positive, and negative faces, and places).

**Reviewer #2:** *This paper reports the results of a data-driven analysis of functional connectivity in the ABCD and HCP datasets. There are many interesting results. Arguably, the main result is that connectome-based predictive models of sustained attention and working memory predict performance in children. However, a youth-specific model of working memory performed better compared to the adult model indicating more developmental differences in the underlying neural correlates. The analyses are based on a well-documented and replicated approach (connectome-based predictive modelling), use large and representative samples, and utilise cross-validation across datasets. The additional control analyses are extensive and very convincing. Further, the discussion sticks closely to the results and provides a well-rounded interpretation. I was only invited as a reviewer after the first round of revisions. I agree with the highly positive assessment of both reviewers regarding the sophisticated methodological approach. I do not agree with the first reviewer's assessment that the study does not add anything to the understanding of neurocognitive development. I think that the authors make a substantial contribution by showing that the functional connectivity correlates of sustained attention and working memory show different developmental patterns, especially because they use a data-driven and unconstrained methodological approach. I think that this study is an absolute triumph and will signify a new standard for conducting research within developmental cognitive neuroscience. In my view, the manuscript can be published without further revisions.*

**Response R2.1.** Thank you for your positive evaluation and time.

**(Very) Minor comments:**

*Figure 6: Please explain what the arrow indicates, either in the caption or in the figure.*

**Response R2.2.** The arrow was a bit confusing and since we do not want it to be misconstrued as developmental change within individuals, we removed it from the manuscript figure. The new figure is included here for convenience.

**Figure 6. The strength of the adult SA network predicts 0-back accuracy in youth and novel adults.** Even though the discriminability of individuals’ task performance is not significantly different within each dataset, i.e., there is no significant difference between the correlations (ABCD = gold, *r =* .19, ρ = .15, *p*s < .001; HCP = violet, *r =* .17, ρ = .12, *ps* < .001; difference between *r*s is not significant Z = .46, *p* =.456), the mean performance and mean network strength are both significantly larger in adults. Overall, adults show stronger sustained attention networks and better 0-back performance than youth (red + signs show the mean of SA strength and 0-back accuracy for the scatterplots in each dataset).

*Figure 7: The difference in correlation with 0-back performance between the youth and adult sample is very difficult to read. Please consider adding some horizontal lines, e.g. at .5 and .75*

**Response R2.3.** We added horizontal lines from the median of the correlation distributions in the PLS LVs to make their corresponding values on the y-axis more readable:
