# Supplementary material for: Differences in the functional brain architecture of sustained attention and working memory in youth and adults
Source: PLoS Biol. 2022 Dec 21;20(12):e3001938. doi: 10.1371/journal.pbio.3001938 (PMC9815648; doi:10.1371/journal.pbio.3001938)
Supplement: Attachment — Submitted filename: Response to Reviews_PlosBioR2.docx [file pbio.3001938.s003.docx]

Thank you for your patience while we considered your revised manuscript "Connectome-based predictions reveal differences in the functional architecture of sustained attention and working memory in youth and adults" for consideration as a Research Article at PLOS Biology. Your revised study has now been evaluated by the PLOS Biology editors and the Academic Editor. As the Academic Editor felt comfortable evaluating the revisions, the revised version was not sent back out to the original reviewers.

**Response 1:** Thank you for your time and constructive feedback.

I am happy to let you know that we are interested in proceeding further with this work at PLOS Biology, with our Academic Editor feeling that the revisions have nicely addressed the prior reviewer concerns and their own concerns. Our Academic Editor does, however, feel that one additional analysis is needed based on some of the new data that has been added.

**Response 2:** Thank you. We have now added analyses to the supplementary section.

Specifically, in the Supplementary Results section (less strict head-motion exclusion criteria in the ABCD) you mention multiple times that comparing the results in the supplementary section with Tables 1-3 in the main text shows no significant changes in the beta coefficients. However, no statistical values (either frequentist or Bayesian) are presented to support this statement, and we feel that such stats are needed. Our Academic Editor suggests that this null hypothesis would be better supported with Bayesian statistics, and encourages you to perform such analyses to make for a more powerful argument. However, if expertise in Bayesian statistics is not something your lab has access to, we ask that you minimally provide further details based on frequentist statistics.

**Response 3:** Thank you for the suggestion. The original concern that led to the less strict head motion analyses was that the brain network strength values may only fit to very low-motion children. So, we used the fact that beta values for more liberal sample did not fall below the confidence intervals around the original regression coefficients to make those statements about similarity. However, we have now conducted a more thorough analysis as suggested by the Academic Editor.

We ran each of the regressions as a Bayesian regression model using *brm()* function in R package ‘brms’ with unspecified priors. These yielded identical regression results to our frequentist models, but included posterior probability distributions for the beta coefficients from 4000 draws of each model. We then quantified non-parametric *p*-values based on comparison of posterior probability distributions of the betas for network strength variables between two tables with the same model but different number of included participants (low-motion with *n* = 1545 and higher motion with *n* = 3225). The results showed that two of the betas were modestly larger in the larger sample (*p* = .025 and *p* = .054), and the other comparisons show non-significant changes (*p*s > .05) (see below). Since no significant decreases were found, our conclusions remain the same. Here are the supplementary tables with the added *p*-value statistics (new text is highlighted):

***Supplementary results with less strict head-motion exclusion criteria*** ***in the ABCD Study sample:***

In addition to the participation weight analysis, we also re-analyzed the data with a less stringent motion threshold of FD mean < .5 mm and FD max < 5 mm to retain more of the ABCD Study sample. The sample size for this FD threshold was n = 3225, which is double the sample with the more stringent FD threshold in the main analyses (FD mean < .2 mm and FD max < 2 mm; n = 1545). Below are the results of the analyses with the .5 mm threshold. The results showed that two of the betas were modestly larger in the larger sample (*p* = .025 and *p* = .054), and the other comparisons show non-significant changes (*p*s > .05) (see below). Since no significant decreases were found, these results echo the same findings from the more restricted sample.

1) Individual differences in sustained attention and working memory network strength are differentially related to individual differences in children’s 0-back (left) and 2-back (right) performance, respectively. Note: Comparing to **Table 1** in the main text with n = 1545 shows no significant changes in the beta coefficient for SA (0-back: *p* = .847, 2-back: *p* = .383 based on Bayesian posterior probability distributions for the betas). The beta coefficient for WM network strength predicting 0-back accuracy is also unchanged (*p* = .565) and it is slightly increased for 2-back accuracy (*p* = .025).

|  | | **0-back Accuracy** | | | |  | **2-back Accuracy** | | | |
| --- | --- | --- | --- | --- | --- | --- | --- | --- | --- | --- |
| *Predictors* | | *Beta* | *CI* | *t* | *p* |  | *Beta* | *CI* | *t* | *p* |
| *(Intercept)* | | 0.03 | [-0.06, 0.12] | 0.68 | 0.499 |  | 0.10 | [0.00, 0.21] | 1.88 | 0.060 |
| *Age* | | 0.13 | [0.10, 0.17] | 7.83 | **<0.001** |  | 0.14 | [0.10, 0.17] | 8.02 | **<0.001** |
| *Female* | | -0.07 | [-0.14, -0.00] | -2.09 | **0.037** |  | -0.21 | [-0.27, -0.14] | -6.29 | **<0.001** |
| *Motion* | | -0.07 | [-0.11, -0.03] | -3.61 | **<0.001** |  | -0.12 | [-0.16, -0.09] | -6.63 | **<0.001** |
| Sustained Attention Network Strength | | 0.14 | [0.10, 0.17] | 7.79 | **<0.001** |  | -0.04 | [-0.07, -0.00] | -2.22 | **0.027** |
| Working Memory Network Strength | | 0.10 | [0.06, 0.16] | 4.19 | **<0.001** |  | 0.14 | [0.11, 0.18] | 7.77 | **<0.001** |
|  | |  |  |  |  |  |  |  |  |  |
| **Random Effects**  **(Site)** | |  |  |  |  |  |  |  |  |  |
|  | ICC | 0.03_site_ | | | |  | 0.04_site_ | | | |
| Observations | | 3225 | | | |  | 3225 | | | |
| Marginal R^2^ / Conditional R^2^ | | 0.076 / 0.103 | | | |  | 0.084 / 0.127 | | | |

**Table S10.** Regression of n-back accuracy against network strength values across ABCD participants with liberal head motion criteria.

2) Block-by-block changes in sustained attention and working memory networks strength values are differentially related to within-subject changes in children’s block-wise 0-back and 2-back performance in children, respectively. Note: Comparing to **Table 2** in the main text with n = 1545 shows no significant changes in the beta coefficients for SA (0-back: *p* = .548, 2-back: *p* = .583) and WM network strengths (0-back: *p* = .673, 2-back: *p* = .381) tracking accuracy values.

|  | | **0-back Accuracy** | | | |  | **2-back Accuracy** | | | |
| --- | --- | --- | --- | --- | --- | --- | --- | --- | --- | --- |
| *Predictors* | | *Beta* | *CI* | *t* | *p* |  | *Beta* | *CI* | *t* | *p* |
| *(Intercept)* | | 0.06 | [0.03, 0.09] | 3.86 | **<0.001** |  | 0.02 | [-0.01, 0.05] | 1.20 | 0.232 |
| *Block Motion* | | -0.06 | [-0.08, -0.05] | -9.16 | **<0.001** |  | -0.06 | [-0.08, -0.05] | -9.30 | **<0.001** |
| *Block Type:* | |  |  |  |  |  |  |  |  |  |
|  | *Negative Face* | -0.10 | [-0.13, -0.08] | -7.08 | **<0.001** |  | -0.01 | [-0.04, 0.02] | -0.62 | 0.537 |
|  | *Positive Face* | -0.01 | [-0.04, 0.02] | -0.47 | 0.635 |  | -0.02 | [-0.05, 0.01] | -1.55 | 0.121 |
|  | *Place* | -0.21 | [-0.24, -0.18] | -14.05 | **<0.001** |  | -0.33 | [-0.36, -0.30] | -22.45 | **<0.001** |
| *Run 2 – Run 1* | | 0.03 | [0.01, 0.05] | 2.70 | **0.007** |  | 0.14 | [0.12, 0.16] | 12.40 | **<0.001** |
| Block Sustained Attention Network Strength | | 0.07 | [0.06, 0.08] | 11.20 | **<0.001** |  | -0.01 | [-0.03, -0.00] | -2.51 | **0.012** |
| Block Working Memory Network Strength | | 0.05 | [0.04, 0.06] | 7.74 | **<0.001** |  | 0.04 | [0.03, 0.05] | 6.16 | **<0.001** |
|  | |  |  |  |  |  |  |  |  |  |
| **Random Effects**  **(Subject)** | |  |  |  |  |  |  |  |  |  |
|  | ICC | 0.41_subs_ | | | |  | 0.42_subs_ | | | |
| Observations (blocks) | | 20999 | | | |  | 21000 | | | |
| Marginal R^2^ / Conditional R^2^ | | 0.022 / 0.422 | | | |  | 0.030 / 0.441 | | | |

**Table S11.** Regression of n-back accuracy against network strength values in blocks within participants using ABCD participants with liberal head motion criteria.

3) Working memory network strength during in-scanner *n*-back task performance is related to subsequent recognition memory for *n*-back task stimuli after adjusting for nuisance variables and even *n*-back performance itself. Note: Comparing to **Table 3** in the main text with n = 1489 shows no significant change in the beta coefficient for WM network strength predicting recognition memory d’ (*p* = .174).

|  | | **Recognition Memory d’** | | | |
| --- | --- | --- | --- | --- | --- |
| *Predictors* | | *Beta* | *CI* | *t* | *p* |
| *(Intercept)* | | 0.64 | [0.59, 0.69] | 24.44 | **<0.001** |
| *Age* | | 0.04 | [0.02, 0.06] | 3.47 | **0.001** |
| *Female* | | 0.04 | [-0.00, 0.08] | 1.89 | 0.059 |
| *Motion* | | -0.02 | [-0.05, 0.00] | -1.82 | 0.069 |
| *n*-back Accuracy | | 0.20 | [0.17, 0.22] | 17.22 | **<0.001** |
| Working Memory Network Strength | | 0.05 | [0.02, 0.07] | 3.93 | **<0.001** |
| Sustained Attention Network Strength | | -0.04 | [-0.06, -0.02] | -3.43 | **0.001** |
|  | |  |  |  |  |
| **Random Effects**  **(Site)** | |  |  |  |  |
|  | ICC | 0.02_site_ | | | |
| Observations  (subjects) | | 3112 | | | |
| Marginal R^2^ / Conditional R^2^ | | 0.125 / 0.143 | | | |

**Table S12.** Regression of recognition memory performance against network strength values across ABCD participants with liberal head motion criteria.

4) Model predicting individual differences in 0-back and 2-back task accuracy from cognitive composite network strength. Note: Comparing to **Table S1** in the supplementary text with n = 1545 shows no significant but marginal increases in the beta coefficient for cognitive composite network strength predicting 0-back (*p* = .081) and 2-back (*p* = .054) accuracy values.

|  | | **0-back Accuracy** | | | | |  | **2-back Accuracy** | | | |
| --- | --- | --- | --- | --- | --- | --- | --- | --- | --- | --- | --- |
| *Predictors* | | *Beta* | *CI* | | *t* | *p* |  | *Beta* | *CI* | *t* | *p* |
| *(Intercept)* | | 0.02 | | [-0.06, 0.10] | 0.38 | 0.703 |  | 0.10 | [0.01, 0.19] | 2.30 | **0.021** |
| *Age* | | 0.13 | | [0.10, 0.16] | 7.57 | **<0.001** |  | 0.11 | [0.08, 0.15] | 7.02 | **<0.001** |
| *Female* | | -0.04 | | [-0.11, 0.02] | -1.24 | 0.214 |  | -0.29 | [-0.25, -0.13] | -5.86 | **<0.001** |
| *Motion* | | -0.09 | | [-0.13, -0.06] | -5.26 | **<0.001** |  | -0.11 | [-0.14, -0.07] | -6.40 | **<0.001** |
| Cognitive Composite | | 0.22 | | [0.19, 0.26] | 12.59 | **<0.001** |  | 0.30 | [0.27, 0.33] | 17.59 | **<0.001** |
|  | |  | |  |  |  |  |  |  |  |  |
| **Random Effects**  **(Site)** | |  | |  |  |  |  |  |  |  |  |
|  | ICC | 0.01_site_ | | | | |  | 0.01_site_ | | | |
| Observations | | 3225 | | | | |  | 3225 | | | |
| Marginal R^2^ / Conditional R^2^ | | 0.093 / 0.111 | | | | |  | 0.154 / 0.177 | | | |

**Table S13.** Regression of n-back accuracy against cognitive composite network strength values across ABCD participants with liberal head motion criteria.

5) The data-driven latent variables (LV) for the sustained attention (left column) and working memory (right column) PLS regressions with mean FD < .5 mm threshold. Note: Comparing to **Figure 7** in the main text with *n* =1545 (mean FD < .2 mm) for the ABCD Study sample shows consistent patterns and no changes in the interpretation of the results (correlations of network patterns between the two analyses: SA LV1: *r* = .992, LV2: *r* = .748, WM LV1: *r* = .914, LV2: *r* = .882; all *p*s <.001). In other words, the primary LVs are still the FC patterns common between youth and adults sample and the second LVs are patterns that are differentially related to performance between youth and adults. In addition, the first LV is still significantly more dominant for the SA PLS compared to the WM PLS and the 2^nd^ LV is more dominant for the WM PLS than SA PLS (*p* < 1/200 for comparison of σ_XY1_ in SA vs. WM or σ_XY2_ in WM vs. SA) despite the difference becoming weaker compared to the lower-motion analysis.

**Figure S9.** PLS regression results including ABCD participants with liberal head motion criteria. The data for this figure are available at NDA study 1849 [10.15154/1528288](http://dx.doi.org/10.15154/1528288).

**IMPORTANT - SUBMITTING YOUR REVISION**
In addition to addressing this statistical issue, when revising the work for resubmission, please address the following points and make sure to carefully address our data and policy issues:

1. Please provide a 'Response to Reviewers' file - this should detail your responses to the additional editorial requests, and indicate the changes made to the manuscript. You should also cite any additional relevant literature that has been published since the original submission and mention any additional citations in your response.

**Response 4:** We added 3 citations to the manuscript, two of which are studies using ABCD task fMRI data and published in 2022 and one is about making circle connectograms. These are highlighted in the document with tracked changes and listed below:

1) Krzywinski, M., Schein, J., Birol, I., Connors, J., Gascoyne, R., Horsman, D., ... & Marra, M. A. (2009). Circos: an information aesthetic for comparative genomics. *Genome research*, *19*(9), 1639-1645.

“**Figure 2**. **Adult sustained attention and working memory networks and their differences.** The circle plots (connectograms) show the sustained attention and working memory networks on the Shen-268 parcels grouped into 20 anatomical regions (10 per hemisphere). The networks positively related to the behavior are shown on the top row and the networks negatively related to the behavior are shown in the bottom row. The matrix plots show the percentage of edges belonging to each macroscale region in working memory connectogram minus the percentage of edges belonging to each macroscale region in sustained attention connectogram. Differences in networks predicting better attention and working memory are shown in the top matrix plot; differences in networks predicting worse attention and working memory are shown in the bottom matrix plot. The data for this figure are available at NDA study 1849 [10.15154/1528288](http://dx.doi.org/10.15154/1528288). Circular plots are made with Circos (Krzywinski et al., 2009).”

2) Chen, J., Tam, A., Kebets, V., Orban, C., Ooi, L. Q. R., Asplund, C. L., ... & Yeo, B. T. (2022). Shared and unique brain network features predict cognitive, personality, and mental health scores in the ABCD study. *Nature communications*, *13*(1), 1-17.

Introduction: “Additionally, validating models of different cognitive processes (e.g., sustained attention and working memory) to evaluate their unique contributions to predicting behavior can determine if distinctions between the models are behaviorally relevant (e.g. see Chen et al., 2022), and whether those distinctions generalize to different stages of development.”

3) Kennedy, J. T., Harms, M. P., Korucuoglu, O., Astafiev, S. V., Barch, D. M., Thompson, W. K., ... & Anokhin, A. P. (2022). Reliability and stability challenges in ABCD task fMRI data. *NeuroImage*, *252*, 119046.

Added methods section:

“***Results are consistent with less motion restricted sample*.** Our main analysis uses a relatively stringent head motion criterion consistent with motion-related reliability issues in the task fMRI in youth (see Kennedy et al., 2022). However, in addition to the participation weight analysis, we also re-analyzed the ABCD data with a less stringent motion threshold of FD mean < .5 mm and FD max < 5 mm to retain more of the ABCD Study sample. The sample size for this FD threshold was *n* = 3225, which is double the sample with the more stringent FD threshold in the main analyses (FD mean < .2 mm and FD max < 2 mm; *n* = 1545). The results are shown in *Supplementary results with less strict head-motion exclusion criteria in the ABCD Study sample* and echo the same findings from the more restricted sample.”

2. In addition to a clean copy of the manuscript, please also upload a 'track-changes' version of your manuscript that specifies the edits made. This should be uploaded as a "Revised Article with Changes Highlighted " file type.

**Response 5:** A document with marked changes in both main manuscript and supplementary results is uploaded, together with clean separate files for main manuscript and supplementary results.

3. Title change: In order to reach our broad readership, when submitting your revision, we'd ask that you consider a slight title change. We'd suggest:
Differences in the functional brain architecture of sustained attention and working memory in youth and adults

OR (though our editorial view is that the method used isn't necessary in the title):
Differences in the functional brain architecture of sustained attention and working memory in youth and adults revealed by connectome-based comparisons

**Response 6:** We changed the title to “Differences in the functional brain architecture of sustained attention and working memory in youth and adults”.

4. IMPORTANT: Please also make sure to address ALL of the following policies and guidelines while preparing your revision:

*PLOS Data Policy*
Please note that as a condition of publication PLOS' data policy (<http://journals.plos.org/plosbiology/s/data-availability>) requires that you make open-access available **all** data used to draw the conclusions arrived at in your manuscript. You therefore must include any data used in your manuscript either in appropriate repositories, within the body of the manuscript, or as supporting information (N.B. this includes any numerical values that were used to generate graphs, histograms etc.). For an example see here: <http://www.plosbiology.org/article/info%3Adoi%2F10.1371%2Fjournal.pbio.1001908#s5>

Note that we **do not** require all raw data. Rather, we ask that all individual quantitative observations that underlie the data summarized in the figures and results of your paper be made available in one of the following forms. The goal is to ensure that our readership has all of the necessary information to recreate the figures in your work. You can provide this information in one of two forms:

1) Supplementary files (e.g., excel). Please ensure that all data files are uploaded as 'Supporting Information' and are invariably referred to (in the manuscript, figure legends, and the Description field when uploading your files) using the following format verbatim: S1 Data, S2 Data, etc. Multiple panels of a single or even several figures can be included as multiple sheets in one excel file that is saved using exactly the following convention: S1_Data.xlsx (using an underscore).

2) Deposition in a publicly available repository. Please also provide the accession code or a reviewer link so that we may view your data before publication. Note that we cannot accept sole deposition of data to GitHub or a similar non-static site (e.g. no personal sites and generally no institutional sites; <https://journals.plos.org/plosbiology/s/data-availability>). Instead, we require deposition to a static site, like Zenodo, FigShare, OSF. GitHub can be used for depositing code however. For your information, note that any data available on a public GitHub site can be directly copied to Zenodo. Once you do this, it will also generate a DOI number that you can provide us with. See the process for doing this here: <https://docs.github.com/en/repositories/archiving-a-github-repository/referencing-and-citing-content>.

Regardless of the method selected, please ensure that you provide the individual numerical values that underlie the summary data displayed in the following figure panels as they are essential for readers to assess your analysis and to reproduce it:
Fig 2,3,4,5,6,7; Fig S1,S3,S4,S5,S6 and final unlabeled graphs in Supp data.

NOTE: the numerical data provided should include all replicates AND the way in which the plotted mean and errors were derived (it should not present only the mean/average values).

**Response 7:** Data for Fig 2, 3, 4, 5, 6, 7; Fig S1, S3, S4, S5, S6, and now labeled graph Fig S9 are all added as separate excel files in the National Institute of Mental Health Data Archive (NDA) study 1849 with doi: [10.15154/1528288](http://dx.doi.org/10.15154/1528288). We cannot share these data on an open access archive due to ABCD Data Use Agreement as per instructions by the NDA help desk that Dr. Kris Dickson is aware of. However, anyone with an NDA account and approved ABCD Data Use Agreement can download these shared data (in addition to other data that are used in the tables and all results in the study). We updated the data availability statement to reflect this.

IMPORTANT: Please also ensure that figure legends in your manuscript include information on where the underlying data can be found, and ensure your supplemental data file/s has a legend. (This step is OFTEN forgotten!!)

**Response 8:** All legends in figures and supplementary figures now have the link to the NDA study where their corresponding data files are located.

Please also ensure that your Data Statement in the submission system accurately describes where your data can be found.

**Response 9:** Here is the updated data and code availability section:

“All ABCD curated data are available at https://nda.nih.gov/edit_collection.html?id=2573. The sustained attention network masks are available at https://github.com/monicadrosenberg/Rosenberg_PNAS2020. The working memory network masks are available per request from the authors of Avery et al. (2020). Specific data used in the analyses of the current study are shared in the National Institute of Mental Health Data Archive (NDA) study 1849 with doi: [10.15154/1528288](http://dx.doi.org/10.15154/1528288). Users with an NDA account and approved Data Use Agreement can access and download the shared data. HCP data are available at <https://db.humanconnectome.org>. Analysis scripts to generate results and figures in the manuscript are available on https://github.com/okardan/ABCD_SA-WM.”

*Ethics Statement – Required
While we note that you’ve indicated an ethics statement is required for your work, we did not see this explicitly mentioned in the methods section of your study. It is entirely possible that we missed this, but please do check and confirm that this statement is, indeed, included.

**Response 10:** We have an ABCD acknowledgement section which should replace the ethics statement. We added the following to the ABCD acknowledgement section and removed the ethics statement indication:

“Secondary analysis of ABCD and HCP data was approved by the relevant University of Chicago Institutional Review Board.”

*Blurb – Required
Please provide a blurb which, if the paper is accepted, will be included in our weekly and monthly Electronic Table of Contents (eTOCs), sent out to readers of PLOS Biology. This blurb may also be used to promote your article on social media. The blurb should be about 30-40 words long and is subject to editorial changes. It should, without exaggeration, entice people to read your manuscript, should not be redundant with the title and should not contain acronyms or abbreviations. For examples, view our author guidelines: <https://journals.plos.org/plosbiology/s/revising-your-manuscript#loc-blurb>

**Response 11:** We added the following blurb:

“Adult functional brain network models predict individual differences and within-person changes in pre-adolescents’ sustained attention and working memory performance. Model generalizability is used to estimate similarities and differences in the neurodevelopment of these cognitive processes.”

*Protocols deposition*
To enhance the reproducibility of your results, we recommend that if applicable you deposit your laboratory protocols in [protocols.io](http://protocols.io/), where a protocol can be assigned its own identifier (DOI) such that it can be cited independently in the future. Additionally, PLOS ONE offers an option for publishing peer-reviewed Lab Protocol articles, which describe protocols hosted on [protocols.io](http://protocols.io/). Read more information on sharing protocols at <https://plos.org/protocols?utm_medium=editorial-email&utm_source=authorletters&utm_campaign=protocols> 

*Published Peer Review*
Please note while forming your response, if your article is accepted, you may have the opportunity to make the peer review history publicly available. The record will include editor decision letters (with reviews) and your responses to reviewer comments. If eligible, we will contact you to opt in or out. Please see here for more details:
<https://blogs.plos.org/plos/2019/05/plos-journals-now-open-for-published-peer-review/>

**Resubmission Checklist**
When you are ready to resubmit your revised manuscript, please refer to this resubmission checklist: <https://plos.io/Biology_Checklist>

To submit a revised version of your manuscript, please go to <https://www.editorialmanager.com/pbiology/> and log in as an Author. Click the link labelled 'Submissions Needing Revision' where you will find your submission record.

We expect to receive your revised manuscript within 1 month. Please email us ([plosbiology@plos.org](mailto:plosbiology@plos.org)) if you have any questions or concerns, or would like to request an extension.  At this stage, your manuscript remains formally under active consideration at our journal; please notify us by email if you do not intend to submit a revision so that we withdraw the manuscript.

Thank you again for your submission to our journal. We hope that our editorial process has been constructive thus far, and we welcome your feedback at any time. Please don't hesitate to contact us if you have any questions or comments.

Sincerely,

Kris

**Response 12:** Thank you again for your time and constructive feedback.
